# Absence of the intracellular lipolytic inhibitor G0S2 enhances intravascular triglyceride clearance and abolishes diet-induced hypertriglyceridemia

**DOI:** 10.1172/JCI181754

**Published:** 2025-03-18

**Authors:** Yongbin Chen, Scott M. Johnson, Stephanie D. Burr, Davide Povero, Aaron M. Anderson, Cailin E. McMahon, Jun Liu

**Affiliations:** 1 Department of Biochemistry and Molecular Biology, Mayo Clinic College of Medicine and Science, Rochester, Minnesota, USA.; 2 Department of Cell Biology, University of Texas Southwestern Medical Center, Dallas, Texas, USA.; 3 Division of Endocrinology, Diabetes, Metabolism, and Nutrition, and; 4 Division of Gastroenterology and Hepatology, Mayo Clinic in Rochester, Rochester, Minnesota, USA.; 5 Department of Developmental Biology, Washington University School of Medicine in St. Louis, St. Louis, Missouri, USA.; 6 Molecular Biology and Genetics Department, Cornell College of Agriculture and Life Sciences, Ithaca, New York, USA.

**Keywords:** Endocrinology, Metabolism, Adipose tissue, Atherosclerosis, Obesity

## Abstract

The interplay between intracellular and intravascular lipolysis is crucial for maintaining circulating lipid levels and systemic energy homeostasis. Adipose triglyceride lipase (ATGL) and lipoprotein lipase (LPL), the primary triglyceride (TG) lipases responsible for these two spatially separate processes, are highly expressed in adipose tissue. Yet the mechanisms underlying their coordinated regulation remain undetermined. Here, we demonstrate that genetic ablation of G0S2, a specific inhibitory protein of ATGL, completely abolished diet-induced hypertriglyceridemia and significantly attenuated atherogenesis in mice. These effects were attributable to enhanced whole-body TG clearance, not altered hepatic TG secretion. Specifically, G0S2 deletion increased circulating LPL concentration and activity, predominantly through LPL production from white adipose tissue (WAT). Strikingly, transplantation of G0S2-deficient WAT normalized plasma TG levels in mice with hypertriglyceridemia. In conjunction with improved insulin sensitivity and decreased ANGPTL4 expression, the absence of G0S2 enhanced the stability of LPL protein in adipocytes, a phenomenon that could be reversed upon ATGL inhibition. Collectively, these findings highlight the pivotal role of adipocyte G0S2 in regulating both intracellular and intravascular lipolysis, and the possibility of targeting G0S2 as a viable pharmacological approach to reducing levels of circulating TGs.

## Abstract 2

We define the role of intracellular lipolytic inhibitor G0S2 in adipose tissue in the regulation of intravascular lipolysis and development of diet-induced hypertriglyceridemia and atherosclerosis.

## Graphical abstract

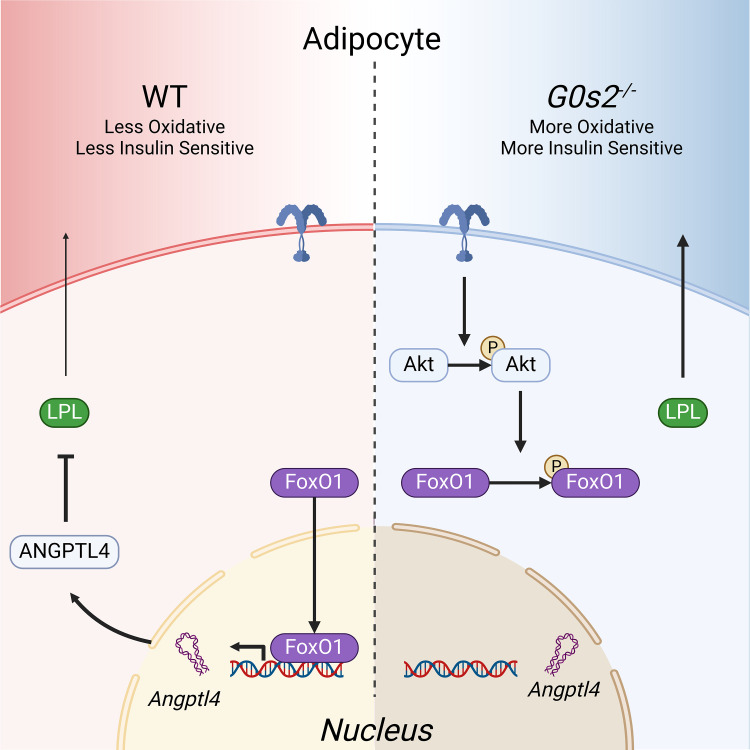

## Introduction

Triglycerides (TGs) represent the largest energy reservoir in the body. White adipose tissue (WAT), the primary TG storage depot, is a dynamic and metabolically active organ that significantly contributes to whole-body lipid and energy homeostasis (1, 2). Upon feeding, adipocytes secrete lipoprotein lipase (LPL), which is involved in the hydrolytic breakdown of circulating TGs into free fatty acids (FAs) and glycerol. Free FAs are subsequently taken up by adipocytes and esterified to form new TGs for storage in intracellular lipid droplets (3, 4). In response to fasting or extended exercise, these TGs are hydrolyzed through the sequential action of adipose triglyceride lipase (ATGL) and hormone-sensitive lipase (HSL), resulting in the release of free FAs and glycerol for utilization by other tissues (5, 6). Thus, TG turnover in adipose tissue is largely mediated by intravascular and intracellular lipolysis through the action of LPL and ATGL. However, the mechanisms underlying the coordinated regulation of these two separate processes remain poorly understood.

LPL is the principal player in the clearance of circulating TG-rich lipoproteins (TRLs) such as chylomicrons and very-low-density lipoproteins (VLDLs) and their remnants. TRLs are arrested at the capillary endothelium by LPL, which forms a complex with glycosylphosphatidylinositol-anchored high-density lipoprotein–binding protein 1 (GPIHBP1) (7). The importance of LPL and GPIHBP1 for plasma TG catabolism in humans is evidenced by the fact that a genetic deficiency in either protein causes a condition of hypertriglyceridemia known as familial chylomicronemia syndrome (FCS) (8). Similarly, mice deficient in LPL or GPIHBP1 are severely hypertriglyceridemic (9, 10). As elevated plasma TG levels are known to be associated with an increased risk of atherosclerotic cardiovascular disease (ASCVD), LPL is generally viewed as an antiatherogenic enzyme (11). However, existing evidence suggests a role for LPL expressed by macrophages in the vessel wall in promoting lipid uptake and foam cell formation (12, 13). Thus, there is a great need to understand the tissue-specific regulation and contribution of LPL to hypertriglyceridemia and atherosclerosis.

The angiopoietin-like (ANGPTL) family of proteins, particularly ANGPTL3, ANGPTL4, and ANGPTL8, act as endogenous antagonists of LPL (14). They are involved in the tissue-specific regulation of LPL under various physiological conditions. In WAT, fasting induces the expression of ANGPTL4, thereby downregulating LPL activity and directing circulating TGs toward oxidative tissues (15–17). Several mechanisms have been proposed to underlie LPL modulation by ANGPTL4 (18, 19). Apart from directly binding and inhibiting LPL extracellularly, ANGPTL4 has been shown to promote intracellular cleavage of LPL in the postendoplasmic reticulum compartment via proprotein convertase subtilisin/kexin type 3 (PCSK3), leading to LPL degradation in adipocytes (20, 21). Genetic ablation of *Angptl4* either globally or specifically in adipose tissue results in enhanced LPL activity, rapid clearance of circulating TGs, and reduced ectopic lipid deposition in liver and muscle (15, 17). This aligns with the observation that ANGPTL4 expression is most abundant in adipose tissue (15–17). Ablation of *Angptl4* in adipose tissue also promotes the clearance of proatherogenic lipoproteins, reduces inflammation, and mitigates atherosclerosis (22). These findings bolster the concept that neutralization of ANGPTL4 has the potential to prevent both hypertriglyceridemia and atherogenesis (23, 24).

ATGL is the rate-limiting enzyme that catalyzes the first step of intracellular lipolysis, converting TG to diacylglycerol (DG) and 1 free FA (6, 25). Previous research conducted by our laboratory and others has established a small protein encoded by g0/g1 switch gene 2 (G0S2) as a potent endogenous inhibitor of ATGL (26–29). Subsequent animal studies have provided compelling evidence demonstrating the role of G0S2 as a major metabolic and energy regulator in adipose tissue, liver, and cardiac muscle through its inhibitory action on ATGL (27, 30–32). In adipocytes, fasting downregulates G0S2 expression, which in turn enhances ATGL-mediated intracellular lipolysis. Consistent with these findings, *G0s2*-deficient (*G0s2^–/–^*) mice preferentially utilize FAs as an energy substrate, and their WAT exhibits enhanced lipolytic activity and increased thermogenic capacity (27, 30–32). In response to a high-fat diet (HFD), these mice remain lean, insulin sensitive, and resistant to weight gain and the development of hepatic steatosis (27, 30, 31).

In the present study, we focused on the metabolic effect of *G0s2* ablation in a mouse model of hypertriglyceridemia and atherosclerosis. Specifically, we investigated the role of G0S2 in the regulation of LPL and ANGPTL4 in adipose tissue. Our data provide evidence that by acting as a switch for both ATGL-mediated intracellular lipolysis and LPL-mediated intravascular lipolysis, G0S2 in adipocytes is critically involved in regulating systemic TG clearance and the development of diet-induced dyslipidemia.

## Results

### G0S2 contributes to diet-induced hypertriglyceridemia but not hypercholesterolemia.

**Figure 1:**
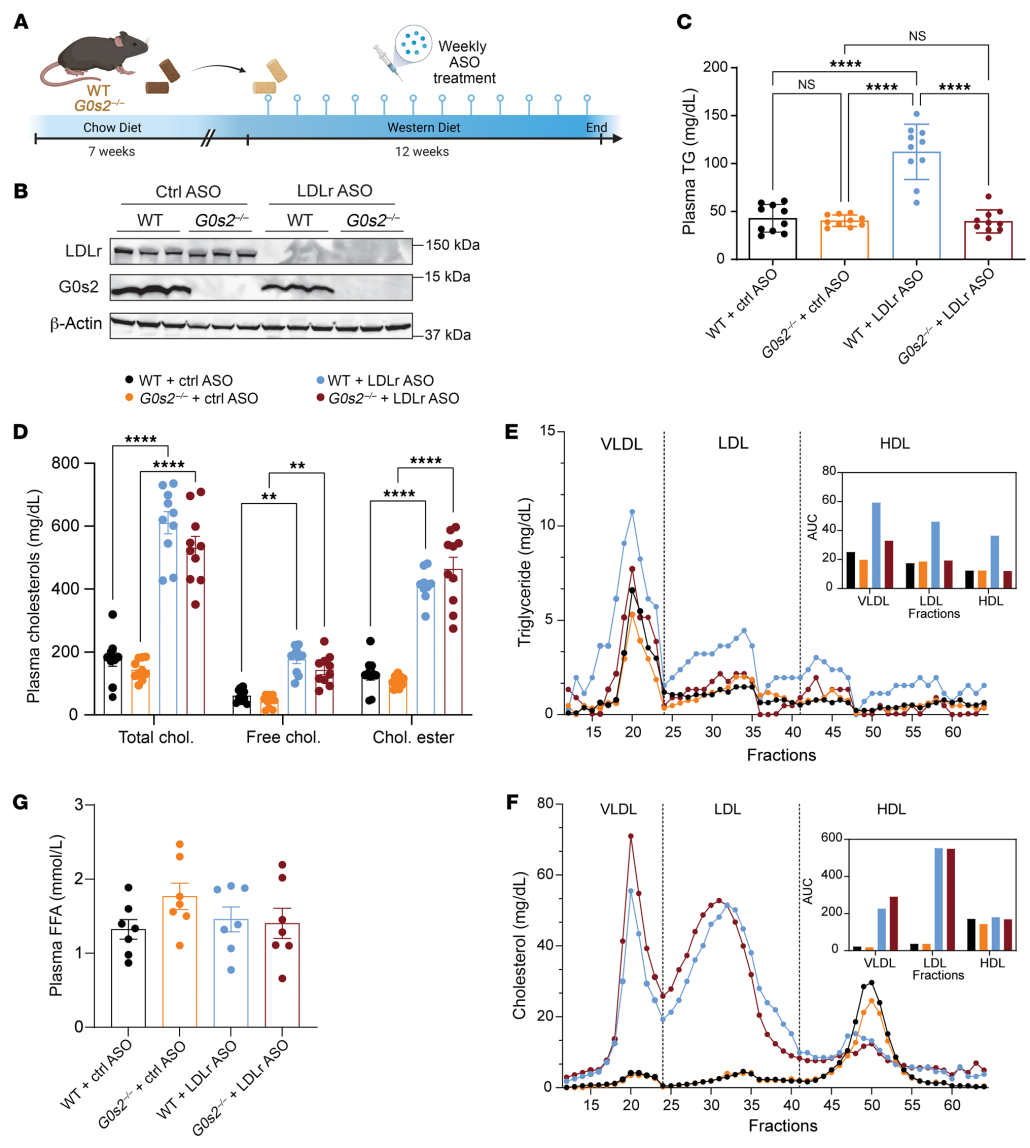
*G0s2* ablation decreases plasma TG in mice treated with Ldlr-ASO and Western diet. (**A**) Mouse model building scheme. Seven-week-old male WT and *G0s2^–/–^* mice were fed a Western diet and treated with either control or Ldlr-ASO weekly for 12 weeks. (**B**) Western blot analysis of mouse liver for LDLR and G0S2. (**C** and **D**) Fasting plasma levels of TG (**C**) and total cholesterol, free cholesterol, and cholesterol ester (**D**) after diet treatment (*n* = 10). Chol., cholesterol. (**E** and **F**) FPLC analysis of pooled plasma TG (**E**) and total cholesterol (**F**) (*n* = 10), with AUC quantification (insets). (**G**) Fasting plasma free FA (FFA) levels (*n* = 7). Data represent mean ± SEM. **P < 0.01, ****P < 0.0001 by 1-way (**C** and **G**) or 2-way ANOVA (**D**).

**Figure 2:**
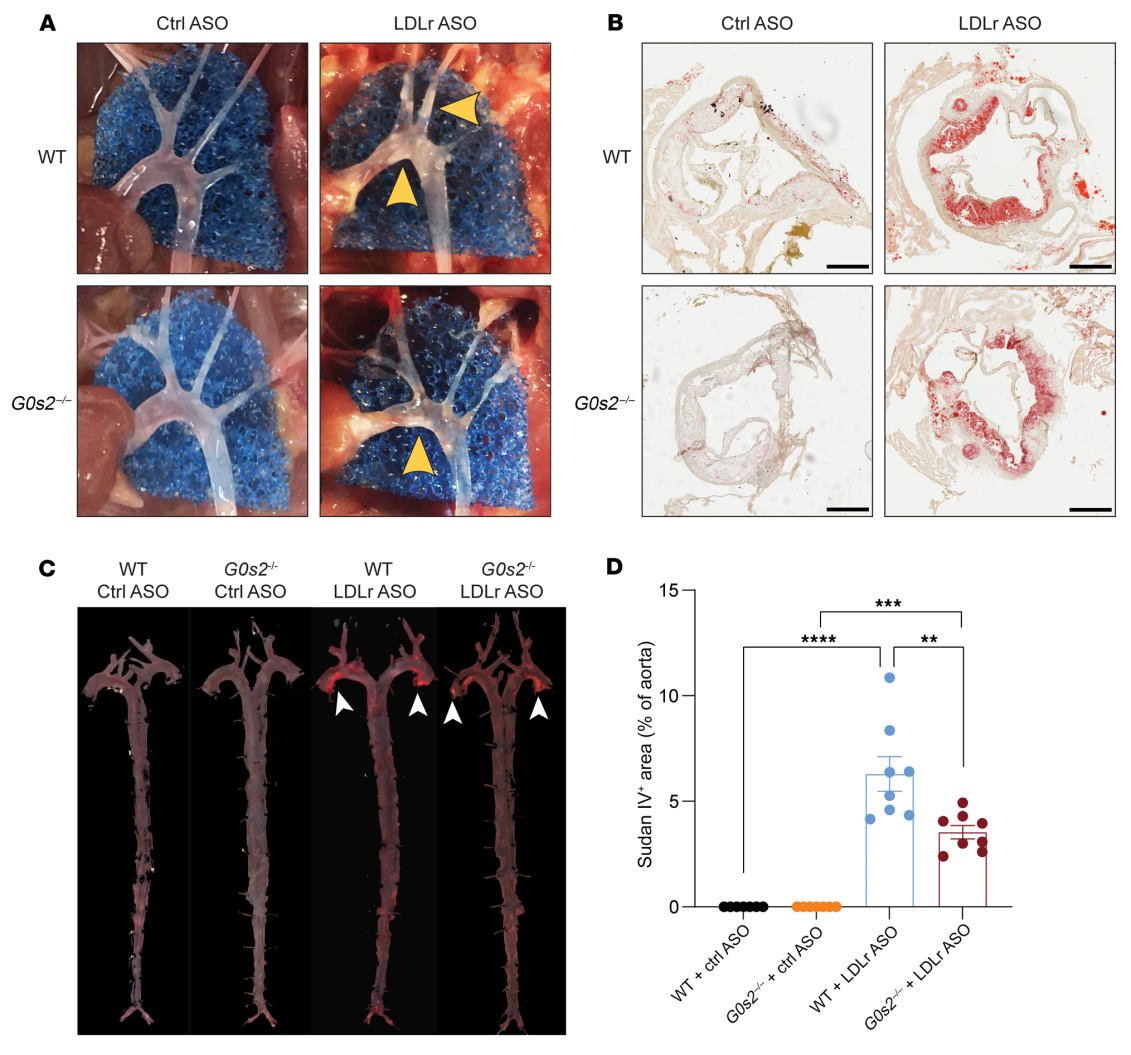
*G0s2* deficiency attenuates atherosclerosis. (**A**) Representative images showing plaque formation (yellow arrowheads) in aortic arch. (**B**) Representative images of plaques in the aortic root sections with Oil Red O staining. Scale bars: 300 μm. (**C**) Representative images of en face Sudan IV–stained aortas with plaques indicated by white arrowheads at aortic roots. (**D**) Quantification of Sudan IV positive areas of total aorta (*n* = 8). Data represent mean ± SEM. **P < 0.01, ***P < 0.001, ****P < 0.0001 by 1-way ANOVA (**D**).

To evaluate the consequence of *G0s2* ablation on diet-induced hyperlipidemia and atherosclerosis, we treated *G0s2^–/–^* mice and their WT littermates with the atherogenic Western diet (21.2% fat, 34% sucrose, and 0.2% cholesterol by weight) for 12 weeks. Concurrently, we injected the mice weekly with either a control antisense oligonucleotide (ASO) or an ASO specifically targeting LDL receptor (Ldlr-ASO) in the liver to create conditions of hypertriglyceridemia and hypercholesterolemia (33) (Figure 1A). At the end of the study, knockdown of hepatic *Ldlr* and knockout of *G0s2* expression were confirmed by Western blot analysis (Figure 1B). In mice receiving the control ASO, no differences in the plasma levels of TG or cholesterol, including total, free, and esterified cholesterol, were observed between the 2 genotypes (Figure 1, C and D). Upon *Ldlr* knockdown, plasma levels of TGs and cholesterol were elevated by 2.6- and 3.6-fold, respectively, in the WT mice. Interestingly, the *G0s2^–/–^* mice receiving Ldlr-ASO were fully protected against the development of hypertriglyceridemia but not hypercholesterolemia (Figure 1, C and D). Specifically, the *G0s2^–/–^* mice exhibited normal fasting plasma TG levels (Figure 1C), as well as a drastic reduction in TG content of all lipoprotein fractions, including VLDL, LDL, and HDL (Figure 1E). In comparison, neither total plasma nor lipoprotein-associated cholesterol content was significantly affected by *G0s2* ablation (Figure 1, D and F), though the size of LDL appeared to increase in the *G0s2^–/–^* mice receiving Ldlr-ASO (Figure 1F). Plasma free FA levels in the 2 genotypes also remained similar (Figure 1G). Furthermore, combinational treatment with Western diet and Ldlr-ASO promoted atherosclerotic development in both WT and *G0s2^–/–^* mice (Figure 2, A–D). However, Oil Red O staining of the aortic root cross-section demonstrated more-pronounced lipid deposition in the WT mice than the *G0s2^–/–^* mice (Figure 2B). En face Sudan IV staining of the entire aorta revealed plaques predominantly localized at the aortic root (Figure 2C). Quantitative analysis of the Sudan IV–positive area indicated that WT mice had a greater total burden of plaques compared with the *G0s2^–/–^* mice (Figure 2D). Taken together, these findings provide evidence that *G0s2* ablation confers resistance against diet-induced hypertriglyceridemia, as well as reducing the severity of atherogenesis without affecting hypercholesterolemia.

### G0s2 ablation decreases adiposity and increases whole-body lipid oxidation.

**Figure 3:**
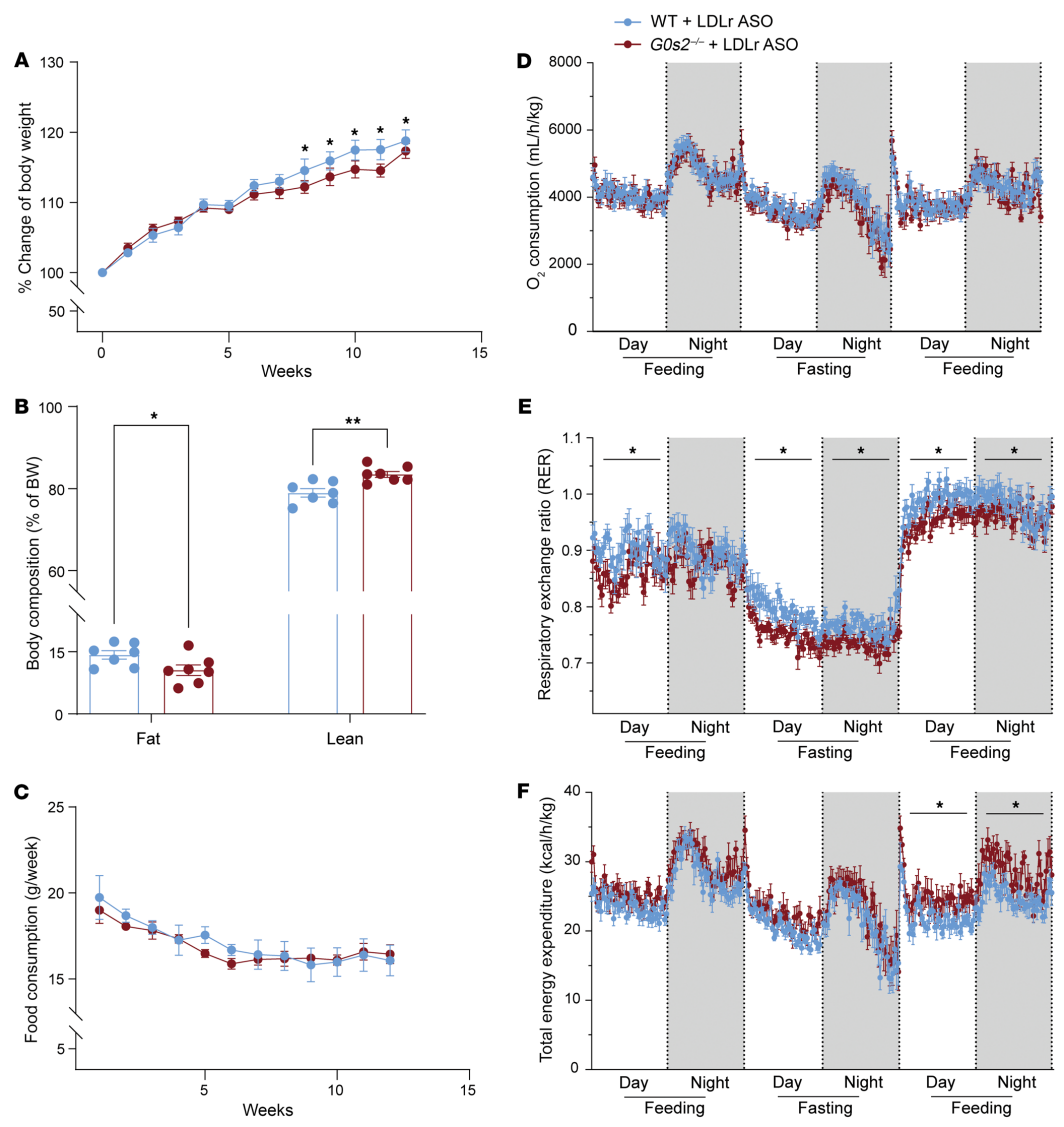
*G0s2* ablation decreases adiposity and increases whole-body lipid oxidation. (**A**) Time course of relative body weight changes in mice treated with Ldlr-ASO and Western diet (*n* = 10). (**B**) Body composition at the end of Ldlr-ASO and diet treatment (*n* = 7). (**C**) Time course of weekly food consumption during diet treatment (*n* = 10). (**D**–**F**) Real-time curve of oxygen consumption (**D**), RER (**E**), and total energy expenditure (**F**) during the feeding/fasting/refeeding cycle (*n* = 10). Data represent mean ± SEM. *P < 0.05, **P < 0.01by unpaired 2-tailed *t* tests (**A** and **C**–**F**) or 2-way ANOVA (**B**). Statistical analysis of metabolic cage data was performed by comparison of the average value of each day or night period.

Along with alleviated hypertriglyceridemia, *G0s2^–/–^* mice showed less body weight gain and a lower percentage of body fat mass than WT littermates when treated with Western diet and Ldlr-ASO (Figure 3, A and B). The difference in body weight gain between the 2 genotypes was abolished as the percentage of lean mass slightly increased in the *G0s2^–/–^* mice. Notably, no significant differences were observed in food consumption between WT and *G0s2^–/–^* mice (Figure 3C). A multiday metabolic cage study showed a similar rate of oxygen consumption during both the fasting and refeeding periods (Figure 3D). However, the respiratory exchange ratio (RER) was shifted significantly downward during fasting and refeeding in *G0s2^–/–^* mice (Figure 3E). In addition, *G0s2^–/–^* mice showed a significant increase in the total energy expenditure in the refeeding period (Figure 3F). These results suggest that *G0s2* ablation promotes overall energy consumption and the preference of lipids to carbohydrates as energy substrate, which is consistent with enhanced ATGL-mediated lipolysis.

### G0s2 ablation improves whole-body TG clearance without affecting hepatic TG secretion.

**Figure 4:**
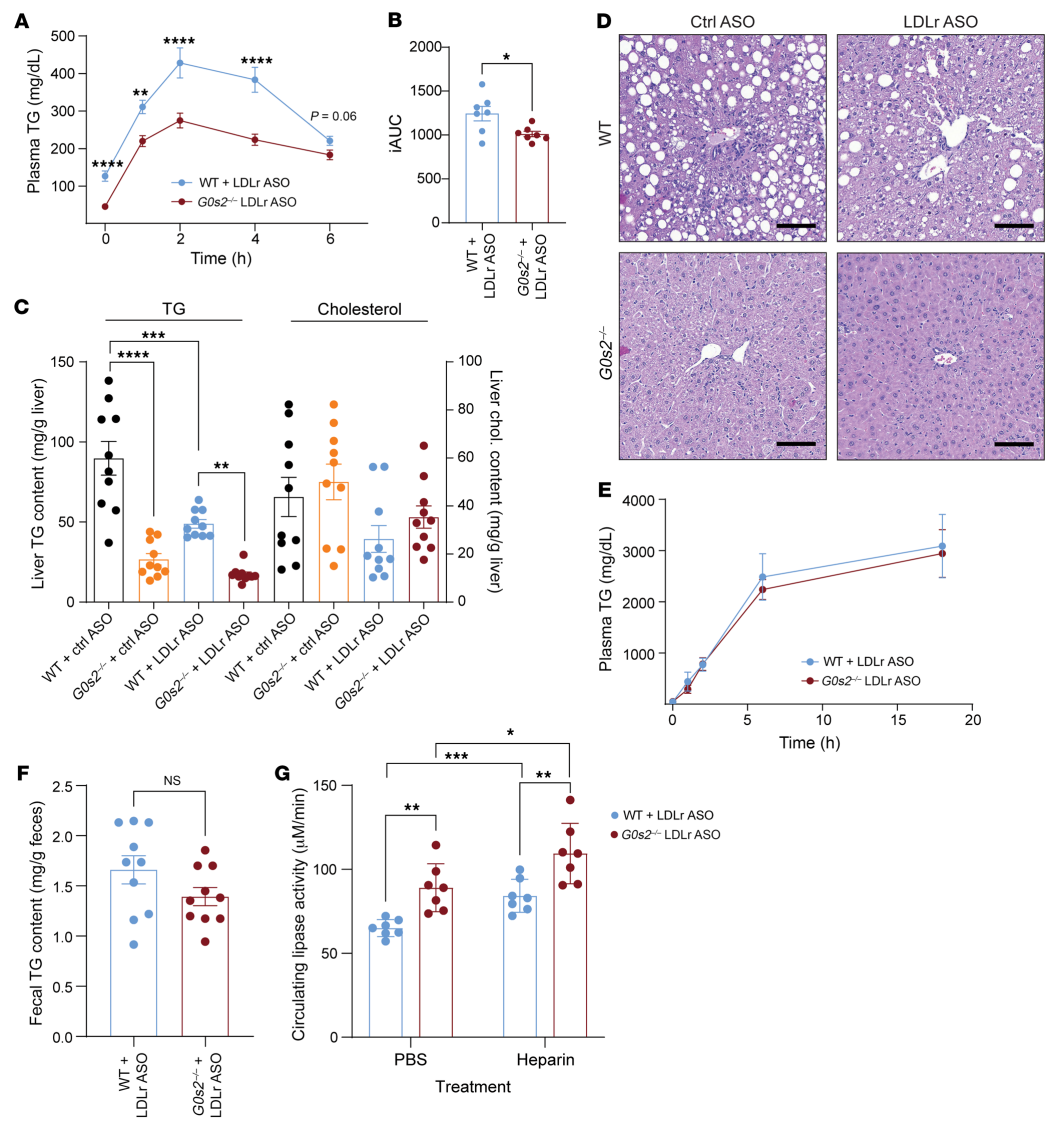
*G0s2* ablation improves whole-body TG clearance without affecting hepatic TG secretion in mice treated with ASO and Western diet. (**A**) Time course of plasma TG levels during OLTT (*n* = 10). (**B**) iAUC analysis of OLTT plasma TG curve (*n* = 10). (**C**) Hepatic TG and cholesterol content (*n* = 10). (**D**) Representative images of liver sections with H&E staining. Scale bars: 100 μm. (**E**) Hepatic TG secretion assay after Poloxamer 407 administration (*n* = 6). (**F**) Fecal TG content (*n* = 10). (**G**) Circulating LPL activity 10 minutes after PBS or heparin administration, as determined following a 15-minute reaction of plasma with an artificial substrate (detailed in Methods). Data represent mean ± SEM. *P < 0.05, **P < 0.01, ***P < 0.001, ****P < 0.0001 by unpaired 2-tailed *t* tests (**A**, **B**, **E**, and **F**) or 2-way ANOVA (**C** and **G**).

To further establish the link between G0S2 and hypertriglyceridemia, we conducted an oral lipid tolerance test (OLTT) in *G0s2^–/–^* mice and their WT littermates. As described above, knockdown of hepatic Ldlr caused a more-pronounced increase in fasting plasma TG levels in WT than *G0s2^–/–^* mice on the Western diet. In the WT mice, an oral gavage of olive oil led to a sharp rise in plasma TG levels, which peaked at the 2-hour time point and then slowly declined over the next 4 hours (Figure 4A). However, in the *G0s2^–/–^* mice, the initial increase was significantly dampened after the 1-hour time point, resulting in a significantly lower peak value at the 2-hour time point (Figure 4A). Comparison of incremental AUC (iAUC) values showed a quantitative improvement in oral lipid tolerance in the *G0s2^–/–^* mice (Figure 4B).

Despite drastically decreased hepatic TG content (Figure 4C) and a loss of lipid vacuoles in liver sections (Figure 4D), *G0s2^–/–^* mice showed no difference from WT mice in the rate of hepatic TG secretion as measured after treatment with Poloxamer 407 (Figure 4E), an LPL inhibitor. Since intestinal lipid absorption as reflected by fecal TG content was also comparable in the 2 mouse lines (Figure 4F), we hypothesized that *G0s2* deficiency would lead to increased plasma TG clearance. To this end, we analyzed TG lipase activity in the plasma. As shown in Figure 4G, *G0s2^–/–^* mice had significantly increased plasma lipase activity both before and after heparin injection. While it was increased by 33.78% prior to heparin injection, post-heparin plasma lipase activity in *G0s2^–/–^* mice was increased by 29.93% compared with that in WT mice (Figure 4G). These data implicate elevated circulating LPL in the alleviation of diet-induced hypertriglyceridemia in *G0s2^–/–^* mice lacking hepatic Ldlr.

### G0s2 ablation increases oral lipid tolerance and LPL production in chow-fed mice.

**Figure 5:**
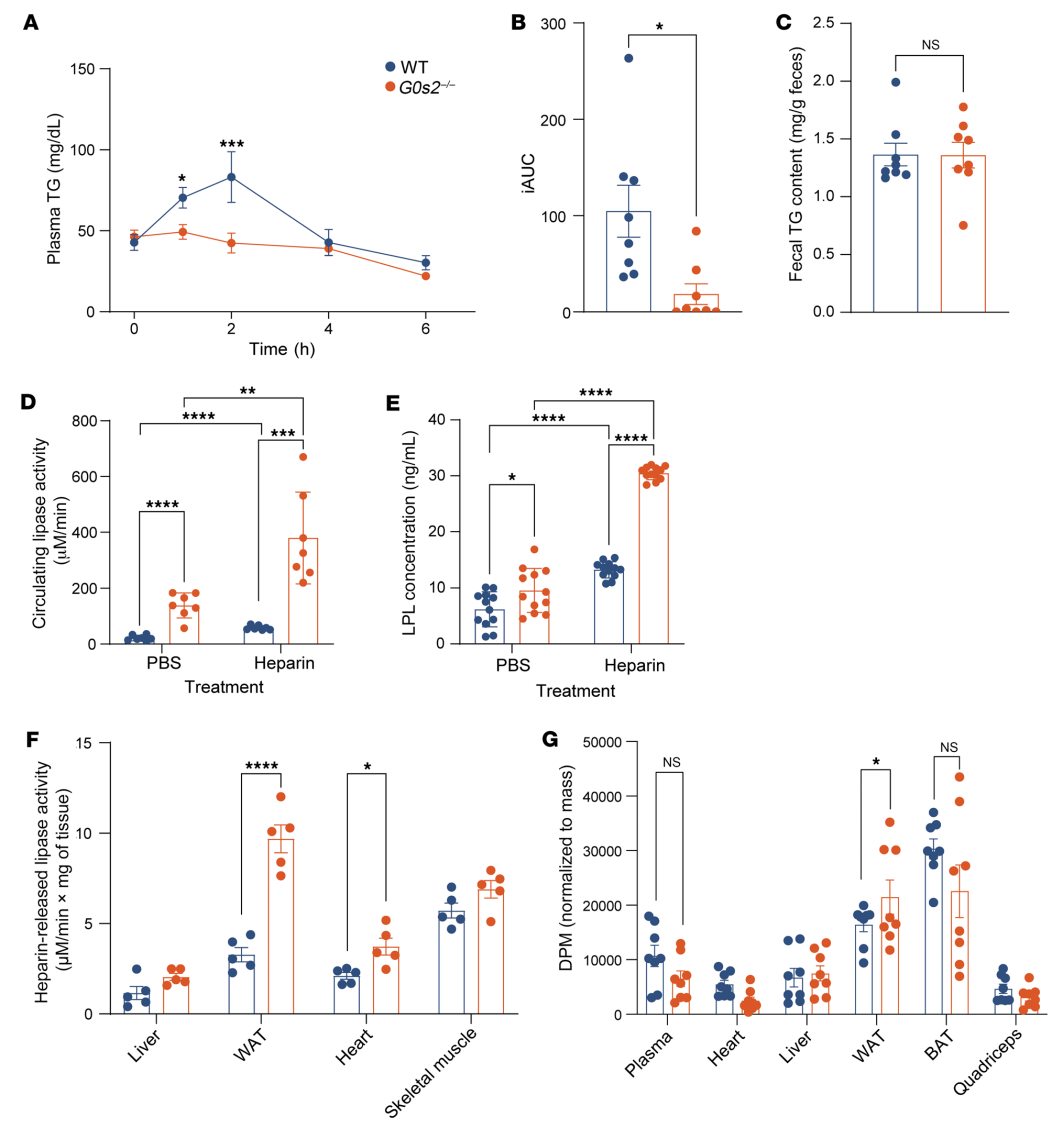
*G0s2* ablation increases oral lipid tolerance and LPL production in chow-fed mice. (**A**) Time course of plasma TG levels during OLTT (*n* = 8). (**B**) iAUC analysis of OLTT plasma TG curve (*n* = 8). (**C**) Fecal TG content (*n* = 8). (**D**) Circulating lipase activity 10 minutes after PBS or heparin administration, as determined following a 60-minute reaction of plasma with the artificial substrate. (**E**) Circulating LPL protein concentration 10 minutes after PBS or heparin administration (*n* = 11). (**F**) Tissue-specific LPL activity (*n* = 5). (**G**) Tissue distribution of ^3^H radioactivity in mice 2 hours after oral gavage of [^3^H]-labeled triolein (*n* = 15). Data represent mean ± SEM. *P < 0.05, **P < 0.01, ***P < 0.001, ****P < 0.0001 by unpaired 2-tailed *t* tests (**A**–**C**) or 2-way ANOVA (**D**–**G**).

Similar to what was observed under the hypertriglyceridemic condition, *G0s2^–/–^* mice fed a chow diet exhibited improved oral lipid tolerance when compared with WT mice (Figure 5, A and B). While no differences in intestinal lipid absorption were observed between WT and *G0s2^–/–^* mice (Figure 5C), plasma lipase activities, both before and after heparin injection, were elevated by more than 6-fold in *G0s2^–/–^* mice (Figure 5D). Plasma concentrations of LPL protein were similarly increased in the *G0s2^–/–^* mice (Figure 5E), suggesting a physiological role for G0S2 in intravascular lipolysis through regulation of LPL production. Measurement of tissue-specific post-heparin lipase activity showed an increase in WAT and heart by 195% and 77%, respectively, upon *G0s2* ablation (Figure 5F). To identify the tissues responsible for the enhanced lipid clearance, we analyzed lipid uptake using a [^3^H]-labeled triolein tracer. Figure 5G shows that *G0s2^–/–^* mice displayed increased lipid uptake mainly in WAT as compared with WT controls. Furthermore, the tissue-specific role of G0S2 was demonstrated in adipose-specific *G0s2*-KO (*G0s2*-AKO) mice, which exhibited improved oral lipid tolerance (Supplemental Figure 1, A and B; supplemental material available online with this article; https://doi.org/10.1172/JCI181754DS1), accompanied by elevated pre- and post-heparin circulating lipase activities and LPL protein levels (Supplemental Figure 1, C and D). Taken together, the results suggest that increased LPL production from WAT likely contributes to enhanced TG clearance in *G0s2^–/–^* mice.

### Transplantation with G0s2^–/–^ WAT alleviates diet-induced hypertriglyceridemia in WT recipient mice.

**Figure 6:**
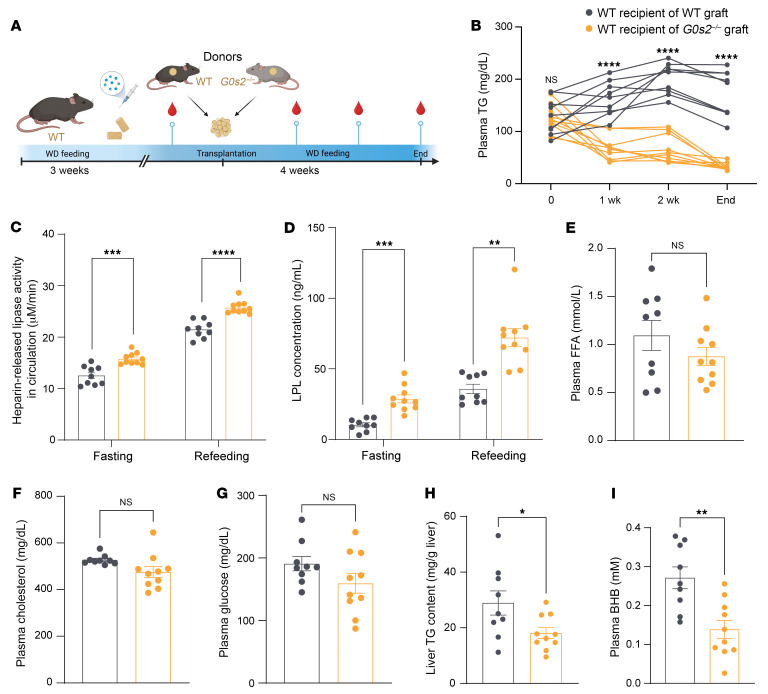
Transplantation with *G0s2*^–/–^ WAT alleviates hypertriglyceridemia in WT recipient mice. (**A**) Experimental scheme: After 3 weeks of treatment with Western diet (WD) and Ldlr-ASO, epididymal WAT dissected from chow-fed WT or *G0s2^–/–^* donor mice was transplanted in situ into WT recipient mice. (**B**) Plasma TG concentrations prior to and after transplantation at the indicated time points (*n* = 9–10). (**C** and **D**) Circulating lipase activity 10 minutes after heparin administration, as determined following a 15-minute reaction of plasma with the artificial substrate (**C**), and LPL protein concentration (**D**) during fasting or refeeding 4 weeks after transplantation (*n* = 9–10). (**E**–**G**) Fasting plasma levels or free FA (**E**), total cholesterol (**F**), and glucose (**G**) at the end point (*n* = 9–10). (**H**) Liver TG content normalized to liver weight at the end point (*n* = 9–10). (**I**). Fasting plasma β-hydroxybutyrate (BHB) levels (*n* = 9–10). Data represent mean ± SEM. *P < 0.05, **P < 0.01, ***P < 0.001, ****P < 0.0001 by unpaired 2-tailed *t* tests (**B**, and **E**–**I**) or 2-way ANOVA (**C** and **D**).

Increased LPL activity in WAT prompted us to examine WAT-specific effect of *G0s2* ablation. Specifically, we tested whether transplantation of WAT from *G0s2^–/–^* mice would improve diet-induced hypertriglyceridemia. To this end, we isolated epididymal fat pads from chow-fed WT or *G0s2^–/–^* mice and transplanted them into WT recipients (Figure 6A). Prior to the experiment, donor mice showed no discernible differences in plasma TG levels (Supplemental Figure 2A). The recipients were pre-fed the Western diet for 3 weeks. At transplantation, the recipients had already developed hypertriglyceridemia, with plasma TG levels elevated by more than 2-fold compared with those in the chow-fed donors (Figure 6B and Supplemental Figure 2A). After WT fat transplantation, the hypertriglyceridemia persisted, with plasma TG continuing to climb and reaching a plateau at the 2-week time point (Figure 6B). In comparison, transplantation with *G0s2^–/–^* fat completely reversed the hypertriglyceridemia, decreasing the plasma TG to levels even below those observed in the chow-fed donors (Figure 6B and Supplemental Figure 2A). Consistent with the normalized TG levels, mice receiving the *G0s2^–/–^* fat graft exhibited significantly higher LPL activity and protein concentration in the plasma than those receiving the WT fat (Figure 6, C and D). The effects appeared to be specific for plasma TG and LPL, as there were no differences in plasma levels of free FA, cholesterol, or glucose between mice transplanted with WT and *G0s2^–/–^* fat (Figure 6, E–G). Body weight and body composition also remained unchanged between the 2 groups (Supplemental Figure 2, B and C). Interestingly, transplantation with *G0s2^–/–^* fat led to a significant reduction in hepatic TG content (Figure 6H). A decreased plasma level of the ketone body β-hydroxybutyrate suggested decreased flux of FAs to liver for oxidation in mice receiving the *G0s2^–/–^* fat (Figure 6I). These results suggest that LPL produced from the *G0s2^–/–^* WAT is a major contributor to the circulating pool in the WT recipients, and that transplantation of *G0s*2*^–/–^* WAT reverses diet-induced hypertriglyceridemia and improves hepatic steatosis.

### G0s2 ablation causes opposite changes in LPL and ANGPTL4 expression in WAT.

**Figure 7:**
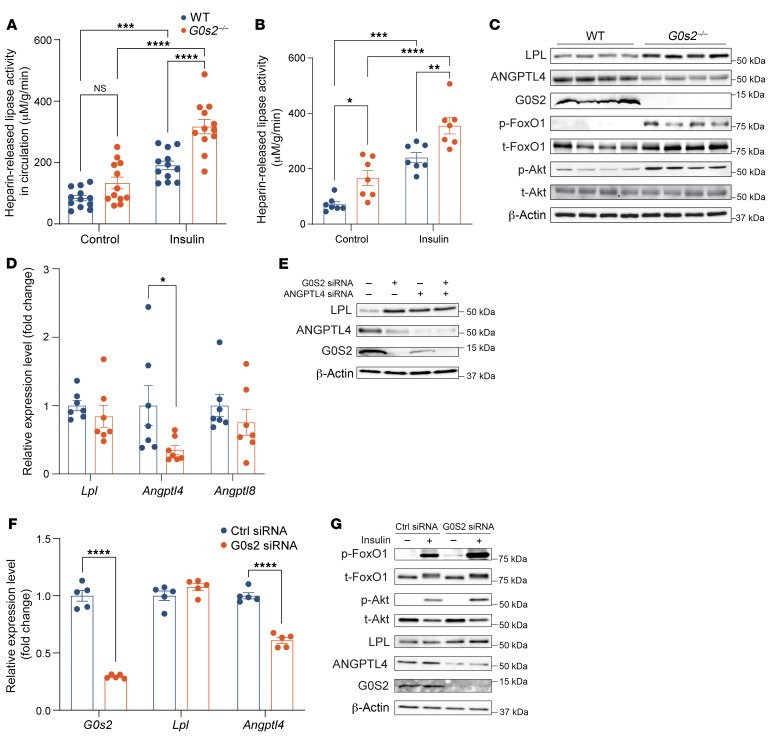
*G0s2* ablation causes opposite changes in LPL and ANGPTL4 expression while increasing insulin sensitivity in adipocytes. (**A**) Circulating post-heparin lipase activity in chow-fed mice before and 1 hour after insulin stimulation (*n* = 12). (**B**) Post-heparin lipase activity in the media of WAT explants without or with insulin stimulation for 45 minutes (*n* = 7). For both **A** and **B**, lipase activity was determined following a 60-minute reaction of plasma with the artificial substrate. (**C**) Western blot analysis of proteins in epididymal WAT after 4-hour refeeding. (**D**) qPCR analysis of relative mRNA expression in epididymal WAT after 4-hour refeeding (*n* = 7). (**E**) Western blot analysis of 3T3-F442A adipocytes transfected with control, G0S2-specific, and/or ANGPTL4 siRNA. (**F**) qPCR analysis of mRNA expression in transfected 3T3-F442A adipocytes. (**G**) Western blot analysis of transfected 3T3-F442A adipocytes after treatment with or without 100 nM insulin for 15 minutes. Data represent mean ± SEM. *P < 0.05, **P < 0.01, ***P < 0.001, ****P < 0.0001 by 2-way ANOVA (**A**, **B**, **D**, and **F**). All cell experiments were conducted 3 times, with similar results, and a representative image is shown.

LPL in WAT is downregulated during fasting and upregulated primarily by insulin during refeeding (34, 35). In comparison to WT mice, *G0s2^–/–^* mice displayed a 59% increase in fasting plasma LPL activity, which was further elevated by 66% upon insulin injection (Figure 7A). Relative to that in WT mice, adipose LPL activity in *G0s2^–/–^* mice was increased by 143% and 48%, respectively, under fasted and insulin-stimulated conditions (Figure 7B). Interestingly, the increases in adipose LPL activity were mirrored by elevations in the expression of LPL protein but not of its mRNA. During refeeding, we observed a significant increase in the expression of LPL protein in WAT of both *G0s2^–/–^* and *G0s2*-AKO mice (Figure 7C and Supplemental Figure 1E), while *Lpl* mRNA levels remained unchanged (Figure 7D and Supplemental Figure 1F). To determine the mechanisms by which LPL protein is regulated in *G0s2^–/–^* WAT, we analyzed expression of 3 ANGPTL family members that are known to posttranslationally downregulate LPL. We observed a significant downregulation of both ANGPTL4 protein and *Angptl4* mRNA in the *G0s2^–/–^* WAT compared with those in WT WAT (Figure 7, C and D, and Supplemental Figure 1, E and F). On the other hand, *G0s2* ablation did not appear to affect expression of *Angptl8* (Figure 7D and Supplemental Figure 1F). By activating Akt-mediated phosphorylation, insulin is known to suppress FoxO1 activity, which can lead to decreased *Angptl4* transcription (36, 37). Consistent with decreased *Angptl4* expression, we observed increased activating phosphorylation of Akt (Ser473), as well as inactivating phosphorylation of FoxO1 (Thr24) in WAT during refeeding (Figure 7C).

To directly test the effects of G0S2 loss on LPL and ANGPTL4, we transfected differentiated 3T3-F442A adipocytes with either a control siRNA or an established siRNA targeting *G0s2* (G0S2-siRNA). As shown in Figure 7, E and F, expression of both *G0s2* mRNA and G0S2 protein was efficiently knocked down in cells receiving the G0S2-siRNA. In the absence of G0S2, cells exhibited increased expression of LPL and decreased expression of ANGPTL4. While changes in ANGPTL4 occurred at both the protein and mRNA levels, G0S2 knockdown affected expression only of LPL protein but not of its mRNA (Figure 7, E and F). In G0S2-knockdown cells, co-knockdown of ANGPTL4 did not further increase LPL protein abundance (Figure 7E), indicating a causal relationship between decreased ANGPTL4 expression and increased LPL abundance in adipocytes lacking G0S2. Furthermore, under the condition of serum deprivation, G0S2 knockdown resulted in an increased response to insulin as shown by Western blot analysis of phospho-Akt and phospho-FoxO1 (Figure 7G). Together, these data demonstrate that *G0s2* ablation led to increased LPL protein expression in adipocytes, which was accompanied by enhanced insulin signaling and decreased expression of ANGPTL4.

### Absence of G0S2 increases LPL protein stability in adipocytes.

**Figure 8:**
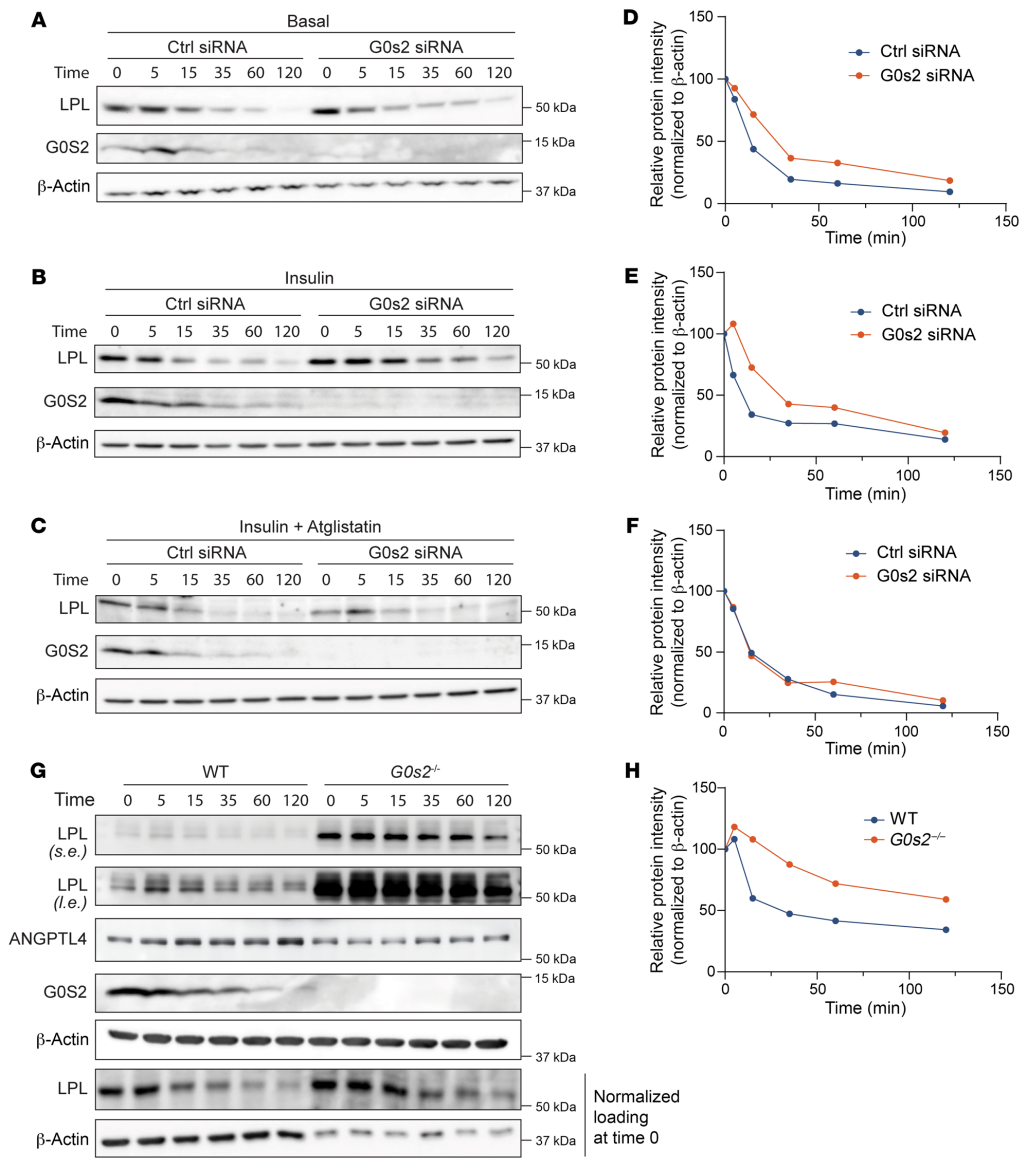
Absence of *G0s2* increases LPL protein stability in adipocytes. (**A**–**C**) Western blot analysis of protein expression in 3T3-F442A adipocytes transfected with either control or G0S2-specific siRNA and then treated with CHX in the presence of vehicle alone (**A**), 100 nM insulin (**B**),or 100 nM insulin + 10 μM Atglistatin (**C**) for the indicated time points. (**D**–**F**) Relative quantification of LPL protein levels in **A** (**D**), **B** (**E**), and **C** (**F**). (**G**) Western blot analysis of SVF-derived adipocytes treated with CHX for indicated periods (s.e., short exposure; l.e., long exposure). (**H**) Relative quantification of LPL intensity in **G**. All cell experiments were conducted 3 times with similar results and a representative image is shown.

As ANGPTL4 is known to promote intracellular degradation of LPL protein (20, 21), we then measured the protein stability of LPL in 3T3-F442A adipocytes in which G0S2 was knocked down. To this end, we treated cells with cycloheximide (CHX), a protein synthesis inhibitor, to determine LPL protein half-life and whether LPL degradation rate is affected by G0S2. Upon CHX treatment, G0S2 knockdown slowed the decay of LPL protein, increasing its half-life from approximately 15 to 30 minutes at basal state (Figure 8, A and D) and from approximately 13 to 32 minutes in the insulin-stimulated condition (Figure 8, B and E). Interestingly, treatment of cells with Atglistatin, a specific chemical inhibitor of ATGL, was able to reverse the degradation rate of LPL in G0S2-knockdown cells to that in the control cells (Figure 8, C and F). Similarly, adipocytes derived from the stromal vascular fraction (SVF) of *G0s2^–/–^* WAT showed increased expression of LPL protein along with decreased expression of ANGPTL4 (Figure 8G). CHX-chase experiments demonstrated enhanced protein stability of LPL in *G0s2^–/–^* cells based on quantitative analysis of protein bands after loading normalization (Figure 8H). Thus, G0S2 inhibition of ATGL-mediated lipolysis played a significant role in the control of LPL protein stability, possibly through upregulation of ANGPTL4 expression.

## Discussion

LPL and ATGL are both highly expressed in adipose tissue. While LPL governs TG turnover intravascularly, ATGL does so intracellularly. Previous studies have identified them as key enzymes that regulate the balance between lipid storage and utilization (3–6). However, the coordinated regulation of LPL and ATGL has remained less defined. In the present study, we uncovered a common mechanism governing their control within adipose tissue. Our findings suggest that G0S2, a known inhibitor of ATGL, acts as a molecular off switch for functional LPL protein production, thereby influencing intravascular lipolysis in adipose tissue and whole-body TG clearance. Furthermore, we provide evidence that this role is linked to the development of hypertriglyceridemia under specific dietary conditions.

Our study highlights the selective role of G0S2 in regulating systemic TG metabolism and its effect on atherosclerosis. The absence of G0S2 conferred resistance to Western diet–induced hypertriglyceridemia in a mouse model with inducible hepatic *Ldlr* knockdown, as evidenced by significantly reduced fasting plasma TG levels across all lipoprotein fractions. Moreover, both global *G0s2^–/–^* and adipose-specific *G0s2*-AKO mice exhibited improved oral lipid tolerance and elevated circulating LPL levels, indicating enhanced TG clearance. Despite the protective effects against hypertriglyceridemia, *G0s2^–/–^* mice still developed atherosclerosis under atherogenic conditions. However, the extent of atherosclerotic progression was significantly reduced compared with that in WT mice, suggesting that *G0s2* ablation partially mitigated disease severity. This underscores the modulatory role of G0S2 in atherogenesis, where its absence reduced, but did not entirely prevent, lesion development. A key innovation in our model is the use of a GalNAc-conjugated ASO that selectively targets hepatic *Ldlr* expression while sparing *Ldlr* in other cell types. This selective approach preserves macrophage LDL receptor function, which may influence atherogenesis. Additionally, the lower TG/HDL ratio observed in *G0s2^–/–^* mice suggests a shift toward larger, less-dense LDL particles. We hypothesize that these larger LDL particles are less readily taken up by macrophages, potentially reducing foam cell formation and slowing the progression of atherosclerosis.

Although G0S2 ablation had no impact on hepatic TG secretion, it enhanced whole-body RER, implying that increased lipid oxidation combined with enhanced TG clearance contributes to mitigating hypertriglyceridemia in these mice. Additionally, the reduction in body weight gain and adiposity observed in *G0s2*-deficient mice align with increased lipolysis and oxidative lipid utilization. These findings further underscore the role of G0S2 in regulation of whole-body lipid metabolism and energy homeostasis. That transplantation of *G0s2^–/–^* WAT completely normalized the circulating TG levels in WT recipients with preexisting hypertriglyceridemia strongly indicates an adipose tissue–autonomous effect of *G0s2* ablation. The elevated circulating levels of LPL in recipient mice indicate that LPL produced by the *G0s2^–/–^* WAT graft was a major contributor to the circulating pool, which presumably accelerated TG catabolism and lipid clearance. This in turn would lead to decreased circulating TG levels and diminished ectopic lipid accumulation in other peripheral tissues. In fact, a significant reduction in hepatic steatosis was observed in mice receiving *G0s2^–/–^* WAT. Given the significant decline in plasma TG without any changes in body weight or fat mass, we speculate that TGs might be cleared through adipose tissue, where FAs derived from LPL action are either efficiently oxidized or stored as TGs that might be further hydrolyzed by ATGL and oxidized, or both in combination. Indeed, prior research has demonstrated heightened lipolysis, increased FA oxidation, and upregulated expression of brown-like genes in WAT of *G0s2^–/–^* mice (27, 30, 31). One study has reported G0S2’s regulatory role in thermogenesis and an augmentation in the expression of FA oxidation genes in the brown adipose tissue (BAT) of *G0s2^–/–^* mice (30). These findings align well with a study involving adipose tissue–specific ATGL-transgenic mice (38). Conversely, in mice with adipose tissue–specific ATGL deficiency, fat mass is preserved due to a decrease in de novo FA synthesis that compensates for the decreased intracellular lipolysis in adipose tissue (39, 40).

An intriguing observation made during the present study is the role of G0S2 in modulating LPL production in WAT. Loss of G0S2 induced heightened intracellular lipolysis facilitated by ATGL. Results from both the *G0s2^–/–^* and *G0s2*-AKO mouse models corroborated an earlier observation of delayed clearance of postprandial plasma TGs resulting from pharmacological inhibition of ATGL (41). While Atglistatin treatment significantly reduced *Lpl* mRNA expression in WAT, G0S2 seems to have exclusively influenced LPL protein expression without affecting its transcription in adipocytes. Mechanistically, the study elucidates that G0S2 inhibition of ATGL-mediated lipolysis played a crucial role in controlling LPL protein stability in adipocytes. In this context, ANGPTL4 is known to inhibit LPL activity through various mechanisms, one of which involves reducing the intracellular stability of LPL protein (20, 21). This occurs through ANGPTL4’s interaction with LPL and induction of its unfolding, thereby increasing the susceptibility of LPL to proteolytic degradation. Consequently, the levels of functional LPL decrease. Thus, it is conceivable that by regulating ANGPTL4 expression and subsequent LPL degradation, G0S2 fine-tunes LPL production and secretion, thereby influencing systemic lipid homeostasis. The fact that co-knockdown of ANGPTL4 did not further increase LPL expression in *G0s2*-knockdown adipocytes supports such a causative relationship. Interestingly, knockout of *Angptl4* in adipose tissue yielded metabolic phenotypes similar to those resulting from *G0s2* ablation, including enhanced LPL activity, rapid clearance of circulating TGs, and increased FA oxidation, thereby preventing excessive ectopic lipid deposition in the liver and reducing hypertriglyceridemia (15, 17, 22).

What is the physiological benefit of G0S2-mediated downregulation of LPL in WAT during feeding? We propose that this regulation ensures that LPL-mediated uptake of TRL-derived FAs coordinates with TG synthesis in white adipocytes, particularly under conditions when WAT is predominantly nonoxidative and geared toward TG storage. By limiting FA uptake through controlled LPL activity, G0S2 may play a role in maintaining WAT homeostasis during feeding. In the absence of G0S2, however, WAT undergoes significant remodeling toward a more insulin-sensitive and oxidative state (27, 30, 31). In this remodeled state, increased LPL production likely facilitates greater FA uptake by adipocytes to support oxidative metabolism. This regulatory shift is accompanied by reduced *Angptl4* expression, which we observed to be linked with enhanced insulin signaling. Specifically, increased phosphorylation of Akt and FoxO1 in adipocytes following refeeding or insulin stimulation indicates heightened insulin responsiveness. Given the established role of FoxO1 in driving *Angptl4* expression (36, 37), we hypothesize that its increased phosphorylation and subsequent inactivation directly contributed to the observed reduction in ANGPTL4 levels. Furthermore, our findings are consistent with studies in adipose-specific ATGL-transgenic mice (38), in which enhanced lipolytic activity in both BAT and WAT promotes mitochondrial FA oxidation, uncoupled respiration, and thermogenic energy dissipation. This metabolic shift alleviates insulin resistance, even under HFD conditions. Collectively, these results highlight the critical role of G0S2 in modulating lipid metabolism, enhancing insulin sensitivity, and promoting metabolic flexibility in WAT.

In summary, the present study offers additional insights into the multifaceted role of G0S2 in lipid and energy metabolism. Understanding the intricate interplay among LPL, ANGPTL4, and G0S2 illuminates the complex regulation of adipose and whole-body TG metabolism. Targeting these regulatory pathways holds promise for developing new therapeutic strategies to manage hypertriglyceridemia and prevent its associated complications. Significantly, our findings that the transplantation of *G0s2^–/–^* WAT alleviated diet-induced hypertriglyceridemia further underscore the therapeutic potential of targeting G0S2 in lipid metabolism disorders.

## Methods

### Sex as a biological variable.

We mainly examined male animals, as the effects of Ldlr-ASO on plasma TG and cholesterol levels as well as atherogenesis are more prominent in male mice (42).

### Animals.

Global *G0s2*-KO mice on a C57BL/6 background were produced as described previously (27). Heterozygous *G0s2*^+/–^ mice were crossed to generate the homozygous *G0s2^–/–^* mice. Seven-week-old male WT and *G0s2^–/–^* littermates were used for experiments. *G0s2*-AKO mice on a C57BL/6 background were generated by crossing homozygous *G0s2* loxP-flanked (*G0s2^fl/fl^*) mice (GemPharmatech Co., strain T013269) with Adipoq-Cre transgenic mice (The Jackson Laboratory, strain 028020). The resulting heterozygous *G0s2^fl/+^* and hemizygous Adipoq-Cre mice were further crossed with homozygous *G0s2^fl/fl^* mice to generate homozygous *G0s2*-AKO mice. The homozygous *G0s2^fl/fl^* littermates were used as WT controls. All mice were housed in the animal facility at Mayo Clinic in Rochester with a 12-hour light/12-hour dark cycle, controlled humidity (40%–70%), and a stable temperature (22°C ± 3°C); and given free access to water and food except when fasting blood specimens were obtained. Mice were fed either a standard chow diet (LabDiet, 5053) or Western diet (Research Diets, D12079B, 0.21% cholesterol), as indicated. Mice and food provided were weighed weekly. Mice were subjected to EchoMRI scanning for body composition analysis. For atherosclerosis model building, 7-week-old WT and *G0s2^–/–^* mice were switched to Western diet and injected i.p. with either a control ASO or a GalNAc-conjugated Gen 2.5 *Ldlr*-ASO (gift from Ionis Pharmaceuticals) at a dose of 5 mg/kg body weight once a week for 12 weeks.

### Protein extraction and Western blot analysis.

Total proteins were extracted from cells or tissues using 1× RIPA buffer (150 mM NaCl, 1% Triton X-100, 0.1% SDS, 0.5% sodium deoxycholate, 1 mM EDTA, and 50 nM Tris pH 7.4) with cOmplete EDTA-free Protease Inhibitor (MilliporeSigma, 11836170001). Protein concentration was determined using a Pierce BCA protein assay kit (Thermo Fisher Scientific, 23225). Total protein was loaded and separated by SDS-PAGE and transferred to a 0.2 μm nitrocellulose membrane (Bio-Rad, 1620112). After blocking membranes in 5% milk in TBST (150 mM NaCl, 15.2 mM HCl, 4.62 mM Tris-Base, 0.1% Tween 20) for 5 minutes at room temperature, primary antibodies diluted in 5% milk were added to the membranes and incubated on a shaker at 4°C overnight. The following rabbit polyclonal or mouse monoclonal primary antibodies were used: anti–mouse LDLR (R&D Systems, AF2255), anti–mouse G0S2 (affinity-purified rabbit polyclonal custom generated by Proteintech Group Inc.) (26), anti–human/mouse LPL (R&D Systems, AF7197), anti–human/mouse ANGPTL4 (Proteintech Group Inc., 67577), anti–mouse FoxO1 (Cell Signaling Technology, 2880), anti–mouse phospho-FoxO1 (Cell Signaling Technology, 2599), anti–mouse Akt (Cell Signaling Technology, 9272), anti–mouse phospho–Akt (Ser473) (Cell Signaling Technology, 9271), anti–β-actin (Santa Cruz Biotechnology Inc., sc-8432). After overnight incubation, membranes were incubated at room temperature for 1 hour with the corresponding goat anti-mouse (Thermo Fisher Scientific, PA186015), goat anti-rabbit (Thermo Fisher Scientific, 65-612-0) or donkey anti-goat (Jackson ImmunoResearch Laboratories, 705-035-147) HRP-conjugated secondary antibodies diluted in 5% milk in TBST. Proteins were visualized by SuperSignal West Pico PLUS Chemiluminescent Substrate (Thermo Fisher Scientific, 34580) and imaged using an ImageQuant LAS4000 instrument (GE Healthcare). Western blot data were collected from a minimum of 3 independent experiments, and a representative membrane is depicted in the figures.

### Blood sampling.

All blood samples were collected after either overnight or 4 hours of fasting. For OLTT and hepatic TG secretion assay, small amounts of blood were collected from submandibular vein. Mice were restrained by grasping over the shoulders. A 25-gauge lancet was used to puncture the vessel at a shallow depth of 1–2 mm. Serial sampling was done using alternate sides of the face. Otherwise, blood samples were collected from retro-orbital plexus of mice using microhematocrit capillary tubes (Thermo Fisher Scientific, 22-362574) and saved in EDTA-coated collectors (RAM Scientific, 076011). Blood was centrifuged at 12,000*g* for 15 minutes for cell precipitation and collection of the plasma. For LPL activity and concentration measurement, freshly collected plasma was used. In other cases, plasma was aliquoted and frozen at –80°C for further experiments.

### Plasma lipid measurement.

Plasma TG was determined by Infinity Triglyceride Reagent (Thermo Fisher Scientific, TR22421), according to the manufacturer’s instructions. An aliquot of each sample was mixed with reagent at 1:100, incubated at 37°C for 5 minutes, and then read by a microplate reader. Plasma cholesterol levels were measured using the Amplex Red Cholesterol Assay Kit (Thermo Fisher Scientific, A12216), according to the manufacturer’s instructions. Each sample was mixed at 1:1 dilution with reaction buffer containing 300 μM Amplex Red reagent, 2 U/mL HRP, 2 U/mL cholesterol oxidase, with or without 0.2 U/mL cholesterol esterase for either total cholesterol or cholesterol ester measurements. After incubation at 37°C for at least 30 minutes, protected from light, the fluorescence was read using excitation between 530 and 560 nm and emission at approximately 590 nm. Free cholesterol was obtained by subtraction of cholesterol ester from total cholesterol. Plasma free FA was assessed by a Wako HR Series Non-Esterified Fatty Acid assay kit (FUJIFILM Wako Chemicals, 997-76491), according to the manufacturer’s instructions. Briefly, each plasma sample was diluted in Color Reagent A at 1:20 and incubated at 37°C for 10 minutes, followed by mixing with Color Reagent B at 2-fold of Reagent A and another 10 minutes of incubation at 37°C. Absorbance at 550 nm of the samples at room temperature was obtained by microplate reader and used to calculate nonesterified FA concentrations. Three replica measurements were performed for each sample group and are reported as mg/dL for TG and cholesterols, and mmol/L for nonesterified FA.

### Fast-protein liquid chromatography.

Fast-protein liquid chromatography (FPLC) analysis was performed at the Analytic Services Core of Vanderbilt Diabetes Research and Training Center. Specifically, 100 μL plasma pooled from 6 mice per group was separated on a Superose 6 column (Amersham Pharmacia Biotech). Forty 0.5 mL fractions were collected. Cholesterol and TG analyses were performed on each fraction using standard enzymatic assays. Fractions 16–24 contained VLDL; 25–44, LDL; and 45–53, HDL. HDL cholesterol was measured with the enzymatic method after precipitation of VLDL and LDL using polyethylene glycol reagent (PEG). From these data, LDL cholesterol was calculated using the Friedewald equation.

### Atherosclerotic lesion analysis.

Each anesthetized mouse was perfused with PBS. The aorta was then exposed, and fat was removed. Pictures of the aortic root and branches were taken using a digital camera. We severed innominate, carotid, and subclavian arteries of aortic arch in thoracic aorta, and iliac arteries in abdominal aorta 3–5 mm after the bifurcations. The aortic root and whole aortas were collected in 10% formalin, kept overnight at 4°C, and then stored in 70% ethanol at 4°C for further processing. For cross-section analysis, the aortic root and ascending aorta were cut and embedded in Tissue-Tek O.C.T. Compound (SAKURA, 4583), frozen, and stored at –20°C. Serial longitudinal cryostat sections (4 μm) were made using a Leica cryostat, mounted on SuperFrost Plus Slides (Cardinal Health, m6146-plus) and stained with Oil Red O. Slides were scanned by a MoticEasyScan Pro 6-FS (Motic). En face analysis of lesions was performed as previously reported. Briefly, the entire aorta was dissected from the proximal ascending aorta to the bifurcation of the iliac artery along the outer curvature until the aortic arch resembled a Y-shaped split. Then the Y-shaped aortas were flattened and pinned onto a black wax petri dish and fixed with 4% buffered paraformaldehyde overnight. On next day, the aortas were washed with 70% ethanol solution twice, followed by staining with Sudan IV staining solution (1% Sudan IV in acetone, diluted with equal volume of 70% ethanol) at room temperature for 10 minutes. Afterward, aortas were briefly washed with 80% ethanol solution 3 times. Images of the stained aortas were taken with PBS filling the black wax petri dish and analyzed with ImageJ software (NIH) to quantify lesion areas. Quantification data are shown as percentage of Sudan IV–positive area normalized to the whole aorta.

### Metabolic cage analysis.

All experimental mice were individually housed in the comprehensive laboratory animal monitoring system equipped with photocells (CLAMS equipped with an Oxymax Open Circuit Calorimeter System; Columbus Instruments) and fed either standard chow diet or Western diet. After a 16-hour acclimation period, metabolic data were continuously collected over a 72-hour period that included 24 hours of ad libitum feeding with the indicated diet, followed by 24 hours of fasting and 24 hours of refeeding. The rate of oxygen consumption (VO_2_) and VCO_2_ values were used to calculate RER; and VO_2_ and RER values were used to determine the basal metabolic rate (kcal/kg/h). Data were normalized to lean body weight per hour and compared between groups by mean of each light or dark cycle.

### OLTT.

OLTT was performed as previously described (43). Briefly, all experimental mice were administered olive oil (100 μL/20 g body weight) by oral gavage, followed by a 4-hour fasting period for clearance of dietary chylomicrons. Submandibular sampling was used to collect plasma right before as well as at the 2-, 4-, and 6-hour time points after olive oil administration. The area under the 6-hour plasma TG curve (AUC) was calculated by use of the trapezium rule. To adjust for the variations in baseline levels, the iAUC was calculated with baseline values subtracted as described previously (44).

### Liver lipid measurement.

Liver extracts were obtained by homogenizing 100–300 mg of liver in 350 μL ethanolic KOH. Homogenates were then incubated overnight at 55°C. Digested tissue was diluted in 1:1 H_2_O/ethanol to a final volume of 1 mL. Samples were microcentrifuged for 5 minutes and supernatant transferred to a new tube. The volume of supernatant was totaled at 1.2 mL with 1:1 H_2_O/ethanol. A volume of 200 μL was transferred to a new tube, where 215 μL of 1 M MgCl_2_ was added. Samples were then vortexed, incubated on ice for 10 minutes, and microcentrifuged for 5 minutes. The supernatant was transferred to a new tube and used for hepatic lipid analysis. TG levels were measured using the Infinity Triglyceride Reagent (Thermo Fisher Scientific, TR22421), and cholesterol levels were determined using the Amplex Red Cholesterol Assay Kit (Thermo Fisher Scientific, A12216), following the manufacturers’ instructions. Liver TG and cholesterol were assessed in each animal of all groups and reported as mg/g liver.

### Histology.

Liver tissue was fixed in 10% formalin, embedded in paraffin (FFPE), and sectioned at 10 μm at the Mayo Clinic Pathology Core. Sections were stained with H&E. Stained slides were imaged by the MoticEasyScan Pro 6-FS.

### In vivo hepatic TG secretion assay.

Hepatic TG secretion was determined by blocking VLDL catabolism using Poloxamer 407 (1 g/kg body weight in PBS, i.p. injection) after a 4-hour fast. Plasma was collected immediately before and at the 1-, 2-, 6-, and 18-hour time points after administration of Poloxamer 407. Plasma TG levels were measured as described above.

### Fecal TG measurement.

Fecal lipids were collected and extracted from mouse feces as described previously (45). Experimental mice were individually housed, with small-particle bedding. Mice were fed ad libitum either a chow diet or Western diet as indicated for 7 days. At the end of study, mice were placed in new cages, and bedding in the old cages was collected for lipid extraction. Feces in old bedding were collected, weighed, and ground into powder. One gram of powdered feces per mouse was used for lipid extraction following the same method applied for liver TG extraction. Fecal TG content is shown as mg/g of feces.

### Lipase activity and LPL concentration analysis.

Mouse plasma was collected 10 minutes after i.p. injection with PBS or heparin (100 U/kg in PBS). In some experiments, epididymal WAT (eWAT) was freshly dissected out of WT and *G0s2^–/–^* mice. The adipose was weighed, cut into small pieces, and immediately put into phenol red–free DMEM for culturing for 30 minutes with or without heparin (50 IU/mL). Media was collected for LPL activity measurement. LPL activity was assessed by an LPL Activity Assay Kit (MilliporeSigma, MAK109), according to the manufacturer’s instructions. 5 μL of each sample (either plasma or cultured media) was mixed with 194 μL LPL Assay Buffer and 1 μL LPL substrate emulsion in a microplate. The plate was sealed and incubated at 37°C for 15–60 minutes. The fluorescence read upon excitation at 370 nm and emission at 450 nm from each sample was used for determination of LPL activity. LPL activity was measured in triplicate and are reported as μM/min for plasma or μM/g · min for eWAT explants.

Plasma LPL concentration was determined by an LPL ELISA kit (MyBioSource, MBS701545). Fresh plasma was collected from WT and *G0s2^–/–^* mice 10 minutes after PBS or heparin administration. Plasma (100 μL) was added into each well and incubated at 37°C for 2 hours. Biotin-antibody (100 μL) was added into each well after removal of sample and incubated for 1 hour at 37°C. The plate was washed with wash solution 3 times, and liquid was completely removed. After addition of 100 μL HRP-avidin to each well, the plate was incubated at 37°C for another 1 hour. TMB Substrate (90 μL) was added after another 5 washes. The plate was subjected to a 30-minute incubation at 37°C. Stop solution was then added at 50 μL/ to each well, and the plate was read at 450 nm for determination of LPL concentrations. Results are reported as ng/mL.

### Tissue isotope tracing assay.

Before the experiment period, WT and *G0s2^–/–^* mice were fed ad libitum from 6:00 pm to 6:00 am and fasted from 6:00 am to 6:00 pm for 5 days to achieve metabolic synchronization. On the day of experiment, mice were fasted for 4 hours to ensure chylomicron clearance and then given 200 μL olive oil containing [^3^H]-labeled triolein (1 μCi) via oral gavage. Liver, heart, epididymal WAT, BAT, and skeletal muscle (quadriceps) were harvested 4 hours after oral gavage, and the specific radioactivity of ^3^H was measured using a liquid scintillation counter. Readings of dpm were normalized to tissue mass.

### Adipose tissue transplantation.

Procedures were performed under isoflurane-induced anesthesia as described previously (46, 47). Briefly, epididymal fat pads were dissected from age-matched, chow-fed male WT and *G0s2^–/–^* mice and transplanted in situ into WT mice pretreated with the Western diet (*n* = 9 WT recipients, *n* = 10 *G0s2^–/–^* recipients). Before transplantation, small incisions were made in the lower abdominal region of anesthetized donor and recipient mice to expose the epididymal fat pads. Donor fat pads were dissected, placed in sterile saline, and cut into 100 mg pieces. These grafts were implanted into the recipient’s epididymal fat pads and secured by sutures. Each recipient received 100 mg donor fat per side (200 mg total). Skin incisions were closed using 5-0 silk sutures. After transplantation, the recipient mice were monitored weekly for plasma TG levels. Four weeks after transplantation and after overnight fasting, tissues and plasma were collected following euthanasia.

### Plasma glucose and β-hydroxybutyrate measurement.

After an overnight fast, blood samples were obtained from the tail vein, and the blood glucose level was measured using a Contour glucometer (Bayer). Fasting plasma β-hydroxybutyrate was determined by a β-Hydroxybutyrate (Ketone Body) Colorimetric Assay Kit (Cayman Chemical, 700190), according to the manufacturer’s instructions. Briefly, each plasma sample was mixed with an equal volume of Developer Solution in each well and incubated at 25°C in the dark for 30 minutes. Absorbance at 450 was used for BHB calculation.

### Cell culture, treatment, and siRNA transfection.

3T3-F442A preadipocytes (MilliporeSigma, CB_ 00070654) were maintained and differentiated as previously described (48). Cells were maintained in DMEM (Gibco, 11995-065) supplemented with 10% newborn calf serum (ATCC, 30-2030) and 1% penicillin/streptomycin (P/S) (Thermo Fisher Scientific, No.15140122) in a humidified atmosphere containing 10% CO_2_ at 37°C. Two days after confluence (day 0), cells were replaced in differentiation media I (DMEM containing 10% FBS (Thermo Fisher Scientific, 10-437-028), 1% P/S, 1 μg/mL insulin (MilliporeSigma, I5523), 0.25 μM dexamethasone (MilliporeSigma, D-4902), and 0.5 mM methyl isobutylxanthine (IBMX; MilliporeSigma, I-7018) for 3 days. Then the media was removed and changed to differentiation media II (DMEM containing 10% FBS and 1 μg/mL insulin). Two days later, the cells were then cultured in DMEM containing 10% FBS for another 2 days. On days 7–8, fully differentiated cells were trypsinized, resuspended in PBS, and subjected to siRNA knockdown via electroporation as described previously (49). Briefly, either 5 μM of an established G0S2-specific siRNA (27) or a GC-matched control siRNA was mixed with 500 μL adipocyte suspension in PBS in a 4 mm electroporation cuvette and pulsed at 950 μF and 160 V by a Bio-Rad Gene Pulser Xcell electroporator. Three days after electroporation, cells were either harvested for protein separation/mRNA extraction or treated with CHX (50 μg/mL), insulin (100 nM), and Atglistatin (10 μM) for the appropriate times.

### Isolation and adipogenic differentiation of mouse SVF cells.

Epididymal fat pads were minced into small pieces and placed in digestion buffer (DMEM supplemented with 1% P/S, 20 mg/mL BSA (MilliporeSigma, A6003), 10 mM CaCl_2_, and 2 mg/mL collagenase (MilliporeSigma, C5138) at 37°C with constant agitation at 150 rpm for approximately 30 minutes. Complete DMEM containing 10% FBS and 1% P/S was then added to stop digestion. The mixture was centrifuged at 700*g* for 10 minutes. The fat layer was discarded, and SVF as the pellet was resuspended in 10% FBS containing DMEM and filtered over a 40 μM strainer. Another centrifugation was then applied to remove death cells and debris. SVF was finally plated on collagen I–coated (Thermo Fisher Scientific, A1048301) plates in complete DMEM. SVF differentiation was induced by replacement of confluent cells with adipogenic medium 2 (high-glucose DMEM containing 10%FBS, 1% P/S, 8 mg/L biotin, 4 mg/L pantothenate, 10 mg/L transferrin,1 mg/L insulin, 0.1 μM hydrocortisone, 0.2 μM triiodo-l-thyronine [T3], 0.25 μM dexamethasone, 0.5 μM IBMX, and 2 μM rosiglitazone). Four days later, the medium was removed, and cells were incubated with DMEM supplemented with 10%FBS, 1% P/S, 8 mg/L biotin, 4 mg/L pantothenate, 10 mg/L transferrin,1 mg/L insulin, 0.1 μM hydrocortisone, and 0.2 μM T3 for another 4–6 days.

### RNA extraction and quantitative real-time PCR analysis.

Total RNA was extracted from cells and tissue using a PureLink RNA Mini Kit (Thermo Fisher Scientific, 12183025) and Ambion TRIZol Reagent (Thermo Fisher Scientific, 15-596-018), respectively, according to the manufacturer’s instructions. cDNA was synthesized from 0.4 μg total RNA using a High-Capacity cDNA Reverse Transcription Kit (Thermo Fisher Scientific, 4368813). Quantitative real-time PCR (qPCR) was performed using Itaq Universal SYBR Green Master Mix (Bio-Rad, 1725124) on a CFX96 Touch Real-Time PCR machine (Bio-Rad) or QuantStudio VII instrument (Life Technologies). Each sample was tested in duplicate or triplicate. To determine the fold change in gene expression compared with a control group, ΔΔCt was calculated. To determine the relative expression of corresponding genes, ΔCt was determined.

### Statistics.

All graphs and statistical analyses were generated using GraphPad Prism 10. Bar graphs represent mean + SEM. *P* values were determined by 2-tailed Student’s *t* test or ANOVA using GraphPad Prism 10. Details regarding statistics are presented in the figures, with legends for *P* values indicated in figure legends. *P* values less than 0.05 were considered significant. Where deemed necessary, NS is included to emphasize that there was no statistical significance; where no value is given, no significance was determined.

### Study approval.

The IACUC at Mayo Clinic approved all animal studies in accordance with federal guidelines. Mayo Clinic IACUC protocol A00005548-20-R23 covering the studies was initially approved on October 7, 2020, and reapproved on September 21, 2023 (expires September 21, 2026).

### Data availability.

All data are available in the main text or the supplemental materials. Values for all data points in graphs are reported in the Supporting Data Values file. Any additional information required to reanalyze the data reported in this paper is available upon request.

## Author contributions

YC designed and performed research, analyzed data, and wrote the manuscript. SMJ, SDB, DP, AMA, and CEM performed research, analyzed data, and edited the manuscript. JL designed and supervised the study as well as writing and editing the manuscript. JL is the guarantor of this work and, as such, had full access to all the data acquired in the study, and takes responsibility for the integrity of the data and the accuracy of the data analysis.

## Supplementary Material

Supplemental data

Unedited blot and gel images

Supporting data values

## Version 1 03/18/2025

In-Press Preview

## Version 2 05/15/2025

Electronic publication
