# Supplementary material for: Absence of the intracellular lipolytic inhibitor G0S2 enhances intravascular triglyceride clearance and abolishes diet-induced hypertriglyceridemia
Source: J Clin Invest. 2025 Mar 18;135(10):e181754. doi: 10.1172/JCI181754 (PMC12077901; doi:10.1172/JCI181754)
Supplement: Supplemental data [file jci-135-181754-s192.pdf]

Supplementary Figure 1. ***G0s2*-AKO mice on chow diet show enhanced lipid tolerance, reduced ANGPTL4 expression and increased LPL production. A.** Plasma TG levels during the oral lipid tolerance test (OLTT) over time (n=7). **B.** iAUC analysis of plasma TG levels during the OLTT (n=7). **C.** Circulating lipase activity measured 10 min after PBS or heparin administration (n=7), as determined following a 60-min reaction of plasma with the artificial substrate. **D.** Plasma LPL protein concentration 10 min post-PBS or heparin administration (n=7). **E**. Western blot analysis of proteins in epididymal WAT after 4-h refeeding following an overnight fast. **F.** Relative mRNA expression in epididymal WAT after the 4-h refeeding (n=7) by qPCR. Data are presented as mean ± SEM. Statistical significance was determined using unpaired two-tailed t-tests (**A&B**) or two-way ANOVA (**C, D&F**): *$p < 0.05$, **$p < 0.01$, ***$p < 0.001$, ****$p < 0.0001$.

Supplementary Figure 2. Impact of WAT transplantation on body weight and compositions. **A**. Plasma TG levels in chow-fed donor mice (WT, n=9; *G0s2*$^{-/-}$, n=10) and WD-fed recipient mice (n=19) prior to transplantation. **B&C**. Total body weight (B) and body compositions (C) before and after transplantation (n=9-10). Data represent mean ± SEM. *$p < 0.05$, **$p < 0.01$, ***$p < 0.001$, ****$p < 0.0001$ by two-way ANOVA (**A, B&C**).

Figure S1

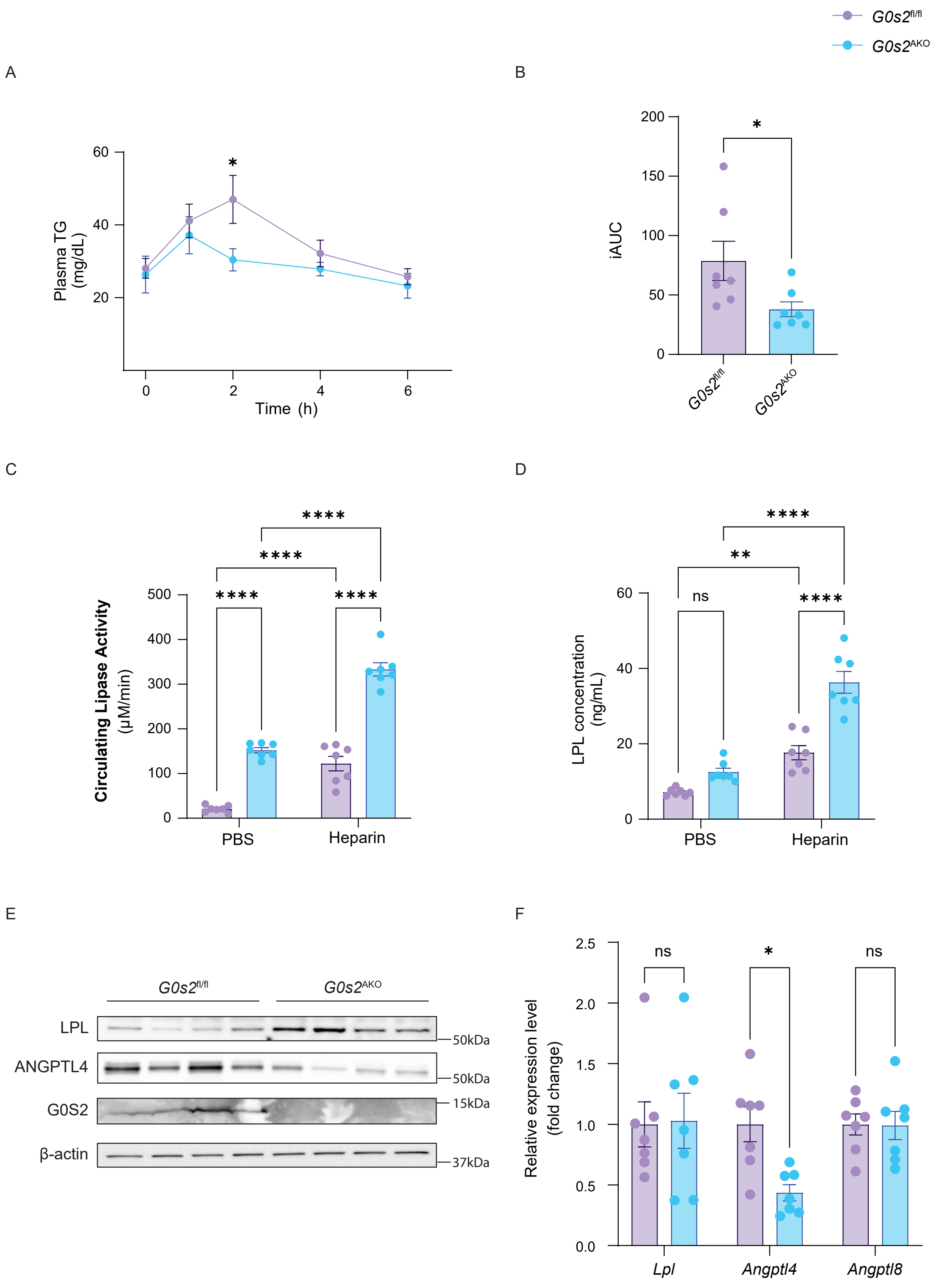

G0s2fl/fl
G0s2AKO
A
Plasma TG (mg/dL)
Time (h)
*
B
iAUC
*
C
Circulating Lipase Activity (μM/min)
****
PBS
Heparin
D
LPL concentration (ng/mL)
ns
**
****
PBS
Heparin
E
G0s2fl/fl
G0s2AKO
LPL
ANGPTL4
G0S2
β-actin
50kDa
50kDa
15kDa
37kDa
F
Relative expression level (fold change)
ns
*
ns
Lpl
Angptl4
Angptl8

Figure S2

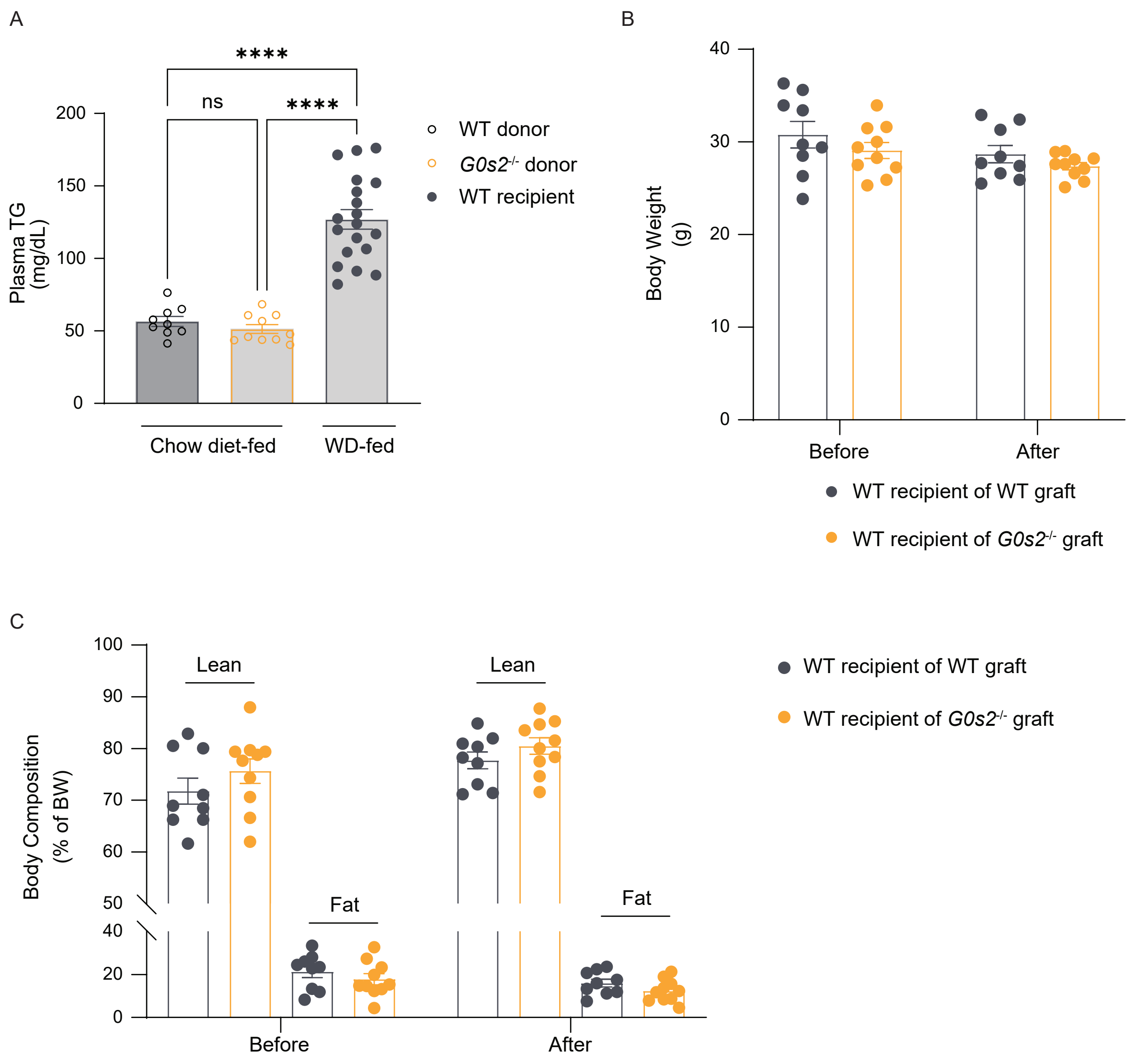

A
****
ns
****
200
150
100
50
0
Plasma TG
(mg/dL)
Chow diet-fed
WD-fed
WT donor
G0s2-/- donor
WT recipient
B
40
30
20
10
0
Body Weight
(g)
Before
After
WT recipient of WT graft
WT recipient of G0s2-/- graft
C
100
90
80
70
60
50
40
20
0
Body Composition
(% of BW)
Lean
Fat
Lean
Fat
Before
After
WT recipient of WT graft
WT recipient of G0s2-/- graft
