# Supplementary material for: Absence of the intracellular lipolytic inhibitor G0S2 enhances intravascular triglyceride clearance and abolishes diet-induced hypertriglyceridemia
Source: J Clin Invest. 2025 Mar 18;135(10):e181754. doi: 10.1172/JCI181754 (PMC12077901; doi:10.1172/JCI181754)

Full unedited gel for Figure 1B

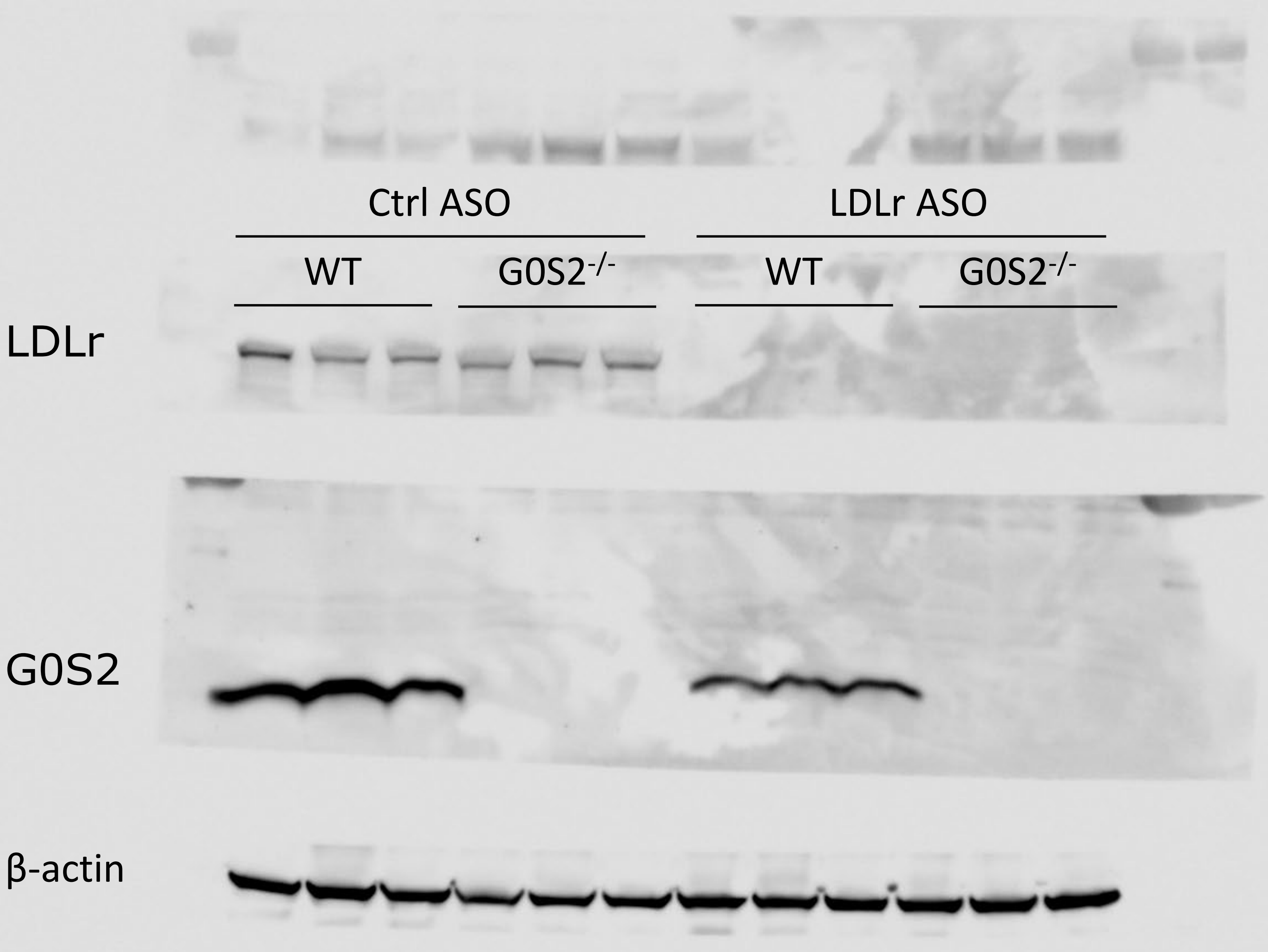

Full unedited gel for Figure 7C

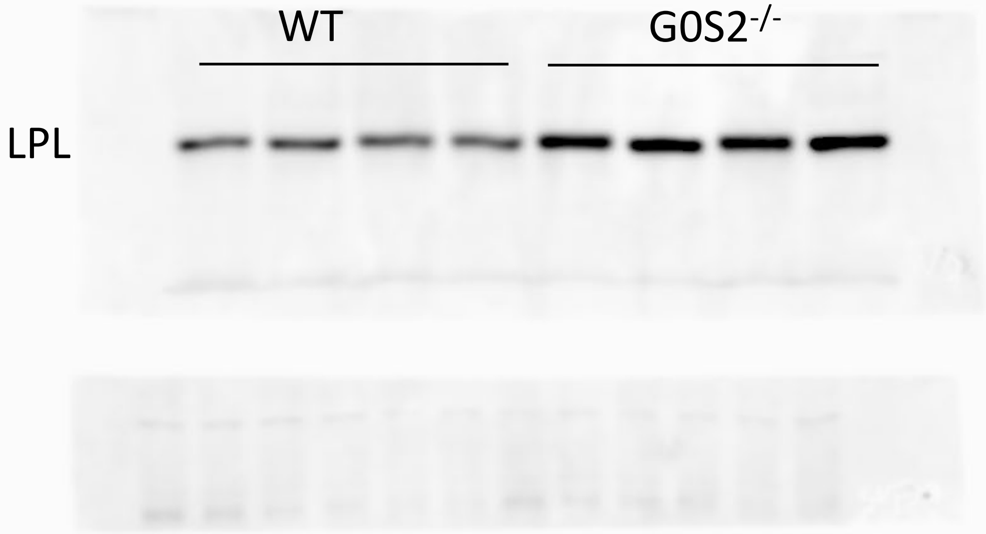

Full unedited gel for Figure 7C

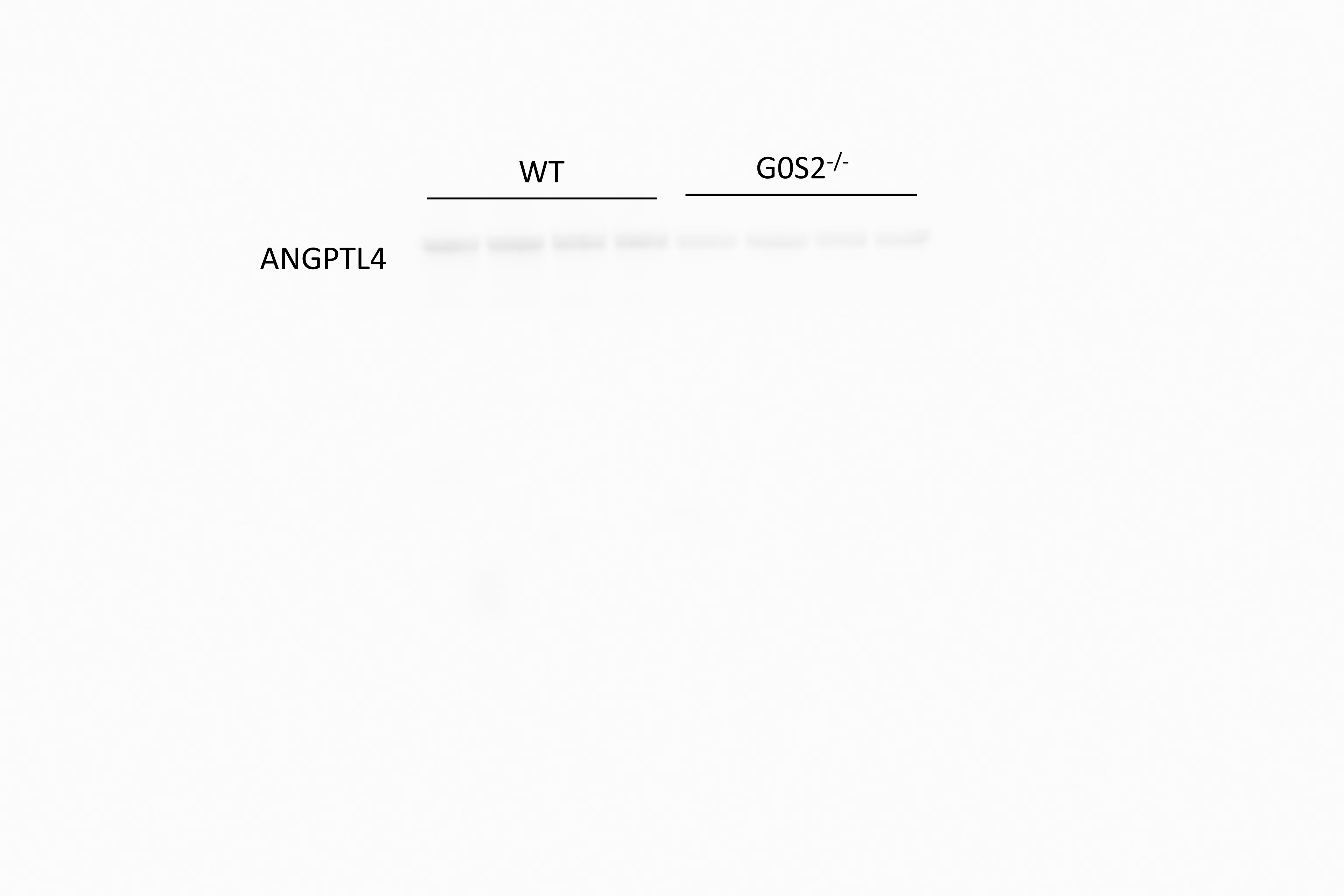

Full unedited gel for Figure 7C

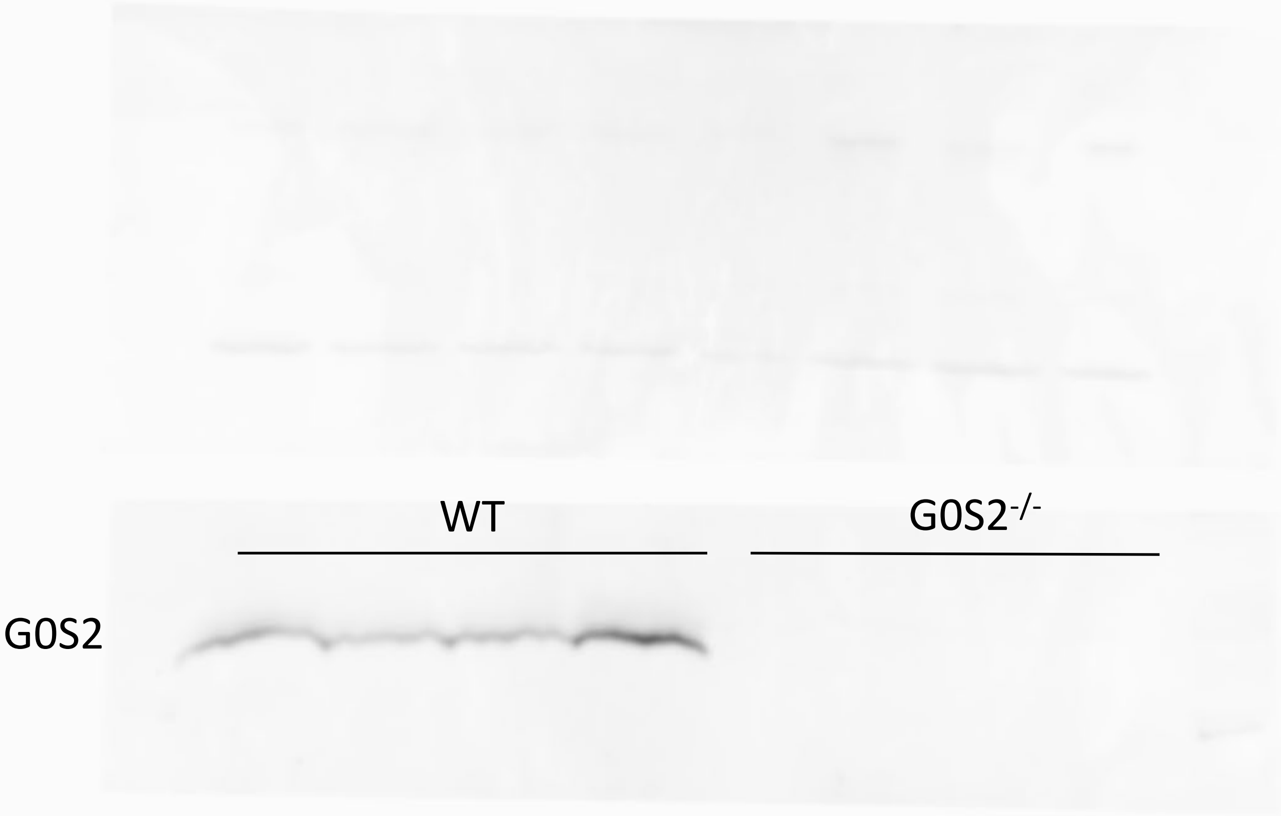

Full unedited gel for Figure 7C

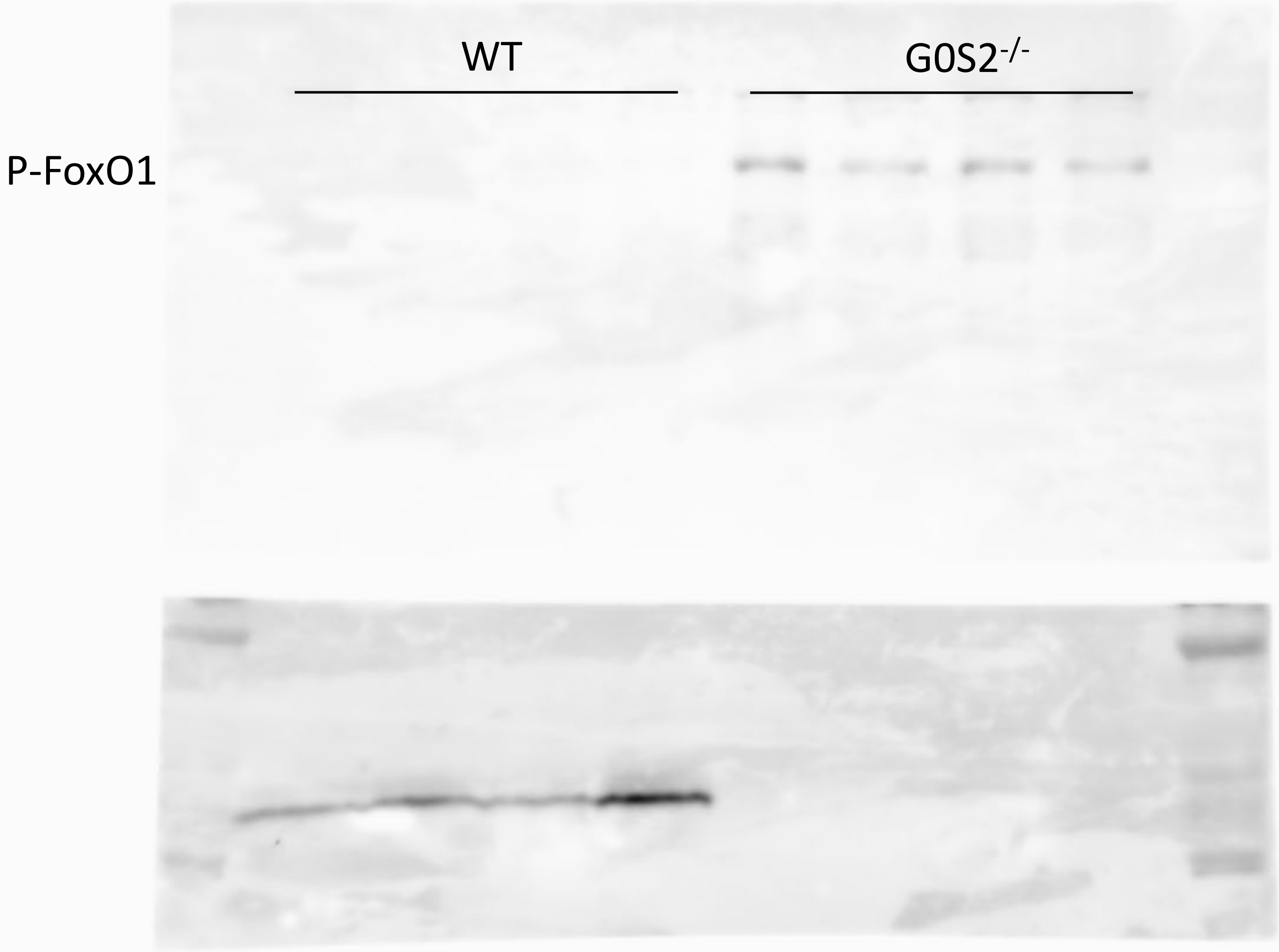

Full unedited gel for Figure 7C

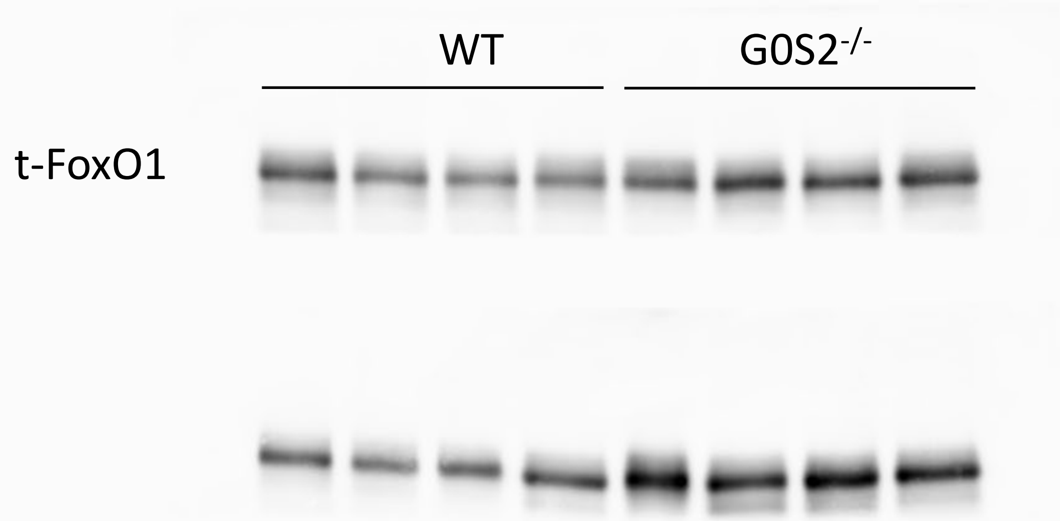

Full unedited gel for Figure 7C

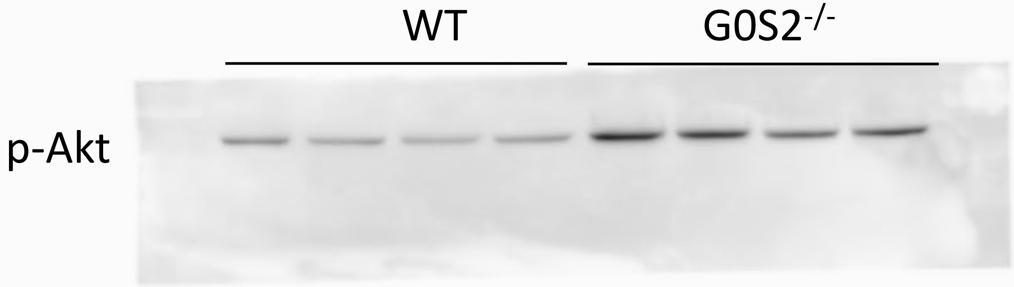

Full unedited gel for Figure 7C

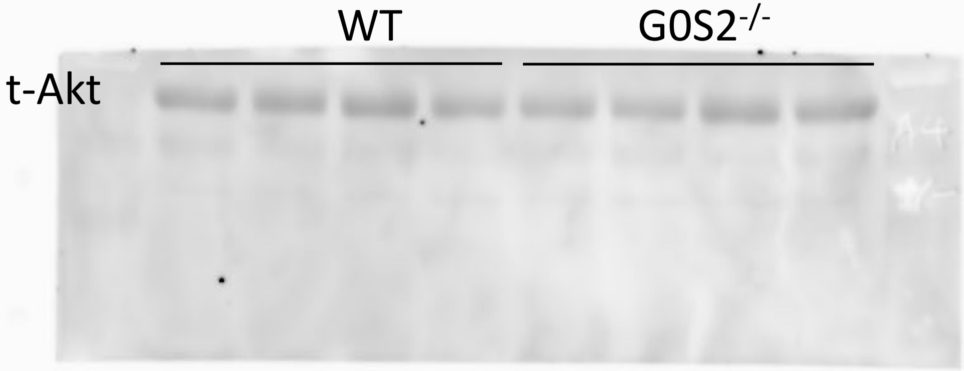

Full unedited gel for Figure 7C

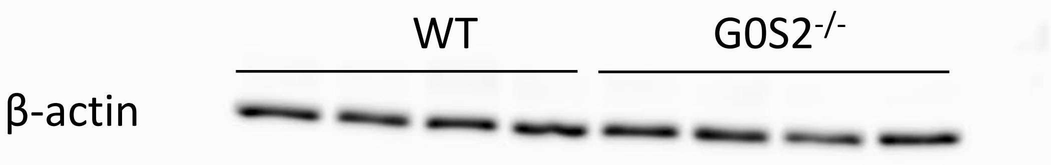

Full unedited gel for Figure 7E

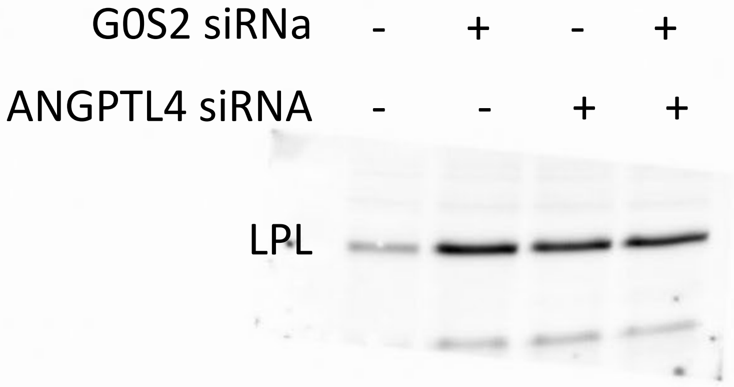

Full unedited gel for Figure 7E

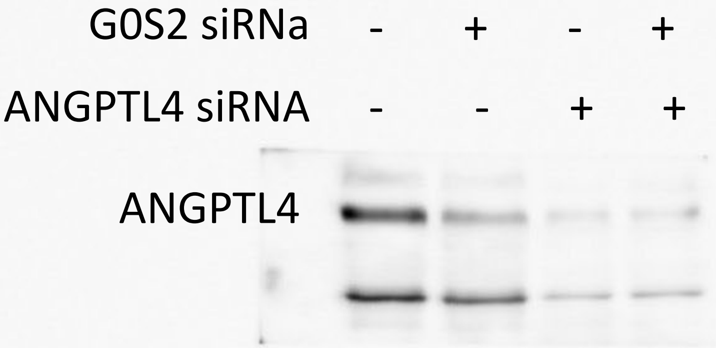

Full unedited gel for Figure 7E

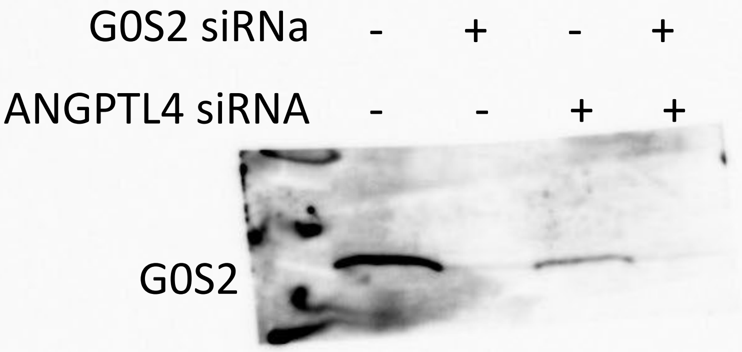

Full unedited gel for Figure 7E

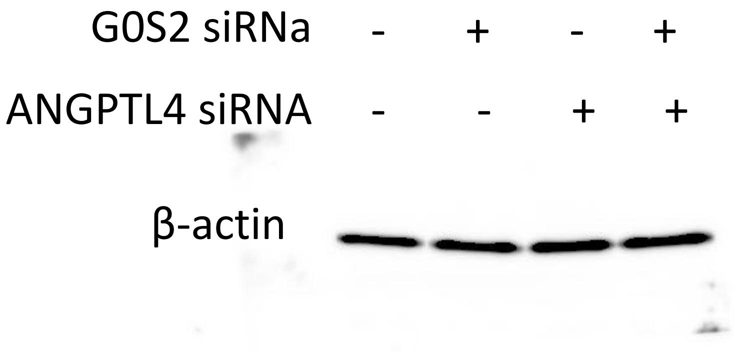

Full unedited gel for Figure 7G

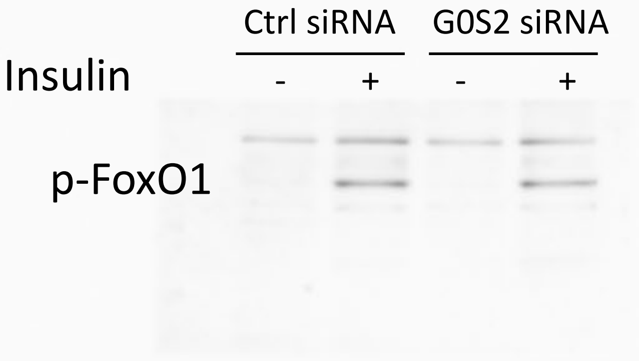

Full unedited gel for Figure 7G

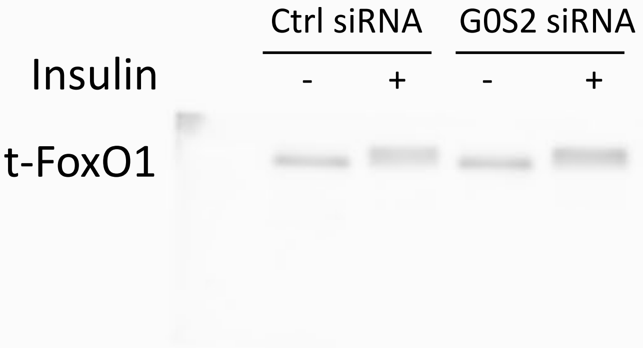

Full unedited gel for Figure 7G

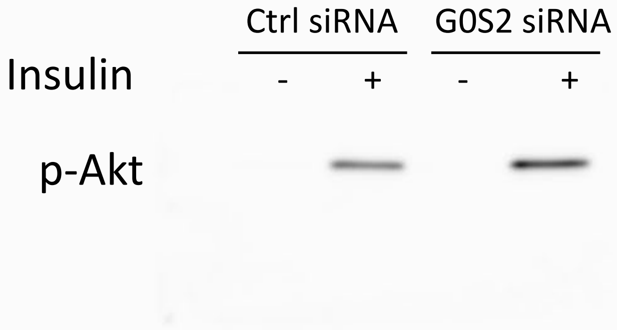

Full unedited gel for Figure 7G

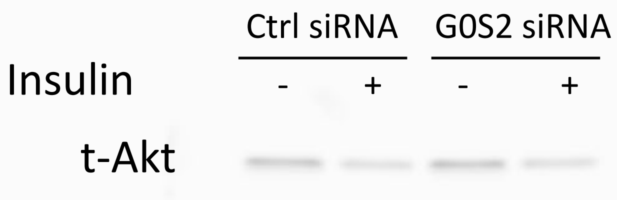

Full unedited gel for Figure 7G

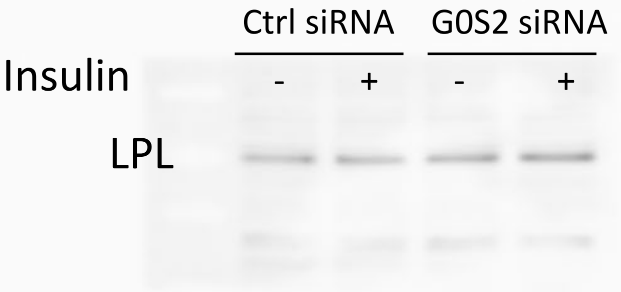

Full unedited gel for Figure 7G

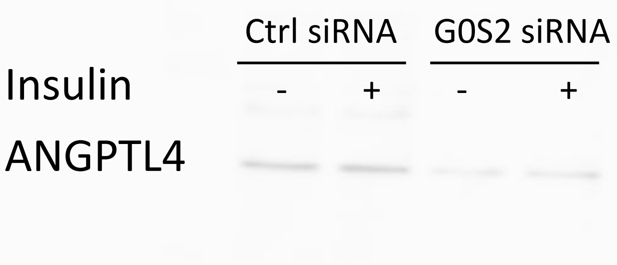

Full unedited gel for Figure 7G

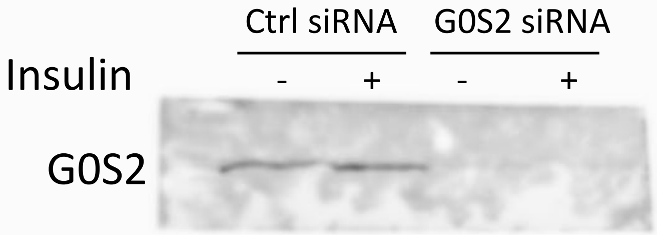

Full unedited gel for Figure 7G

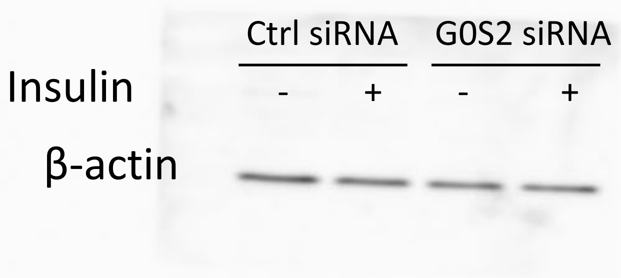

Full unedited gel for Figure 8A

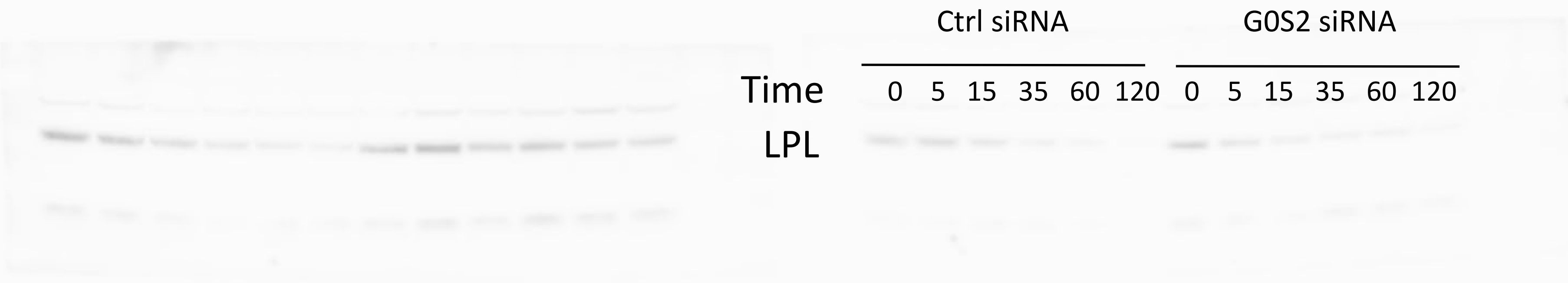

Full unedited gel for Figure 8A

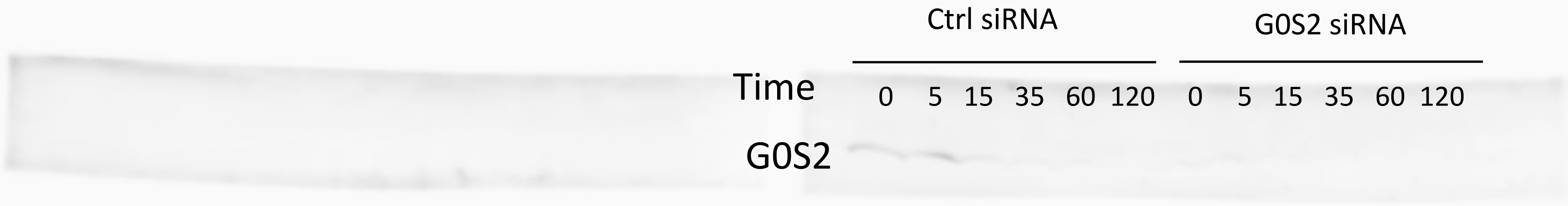

Full unedited gel for Figure 8A

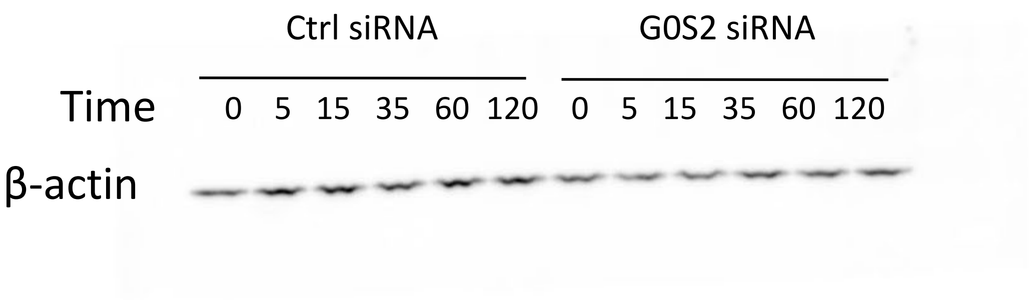

Full unedited gel for Figure 8B

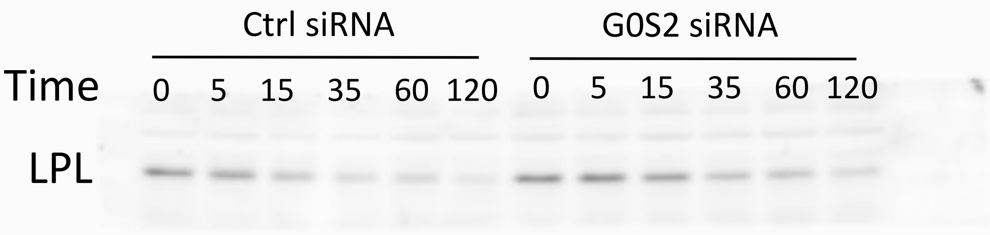

Full unedited gel for Figure 8B

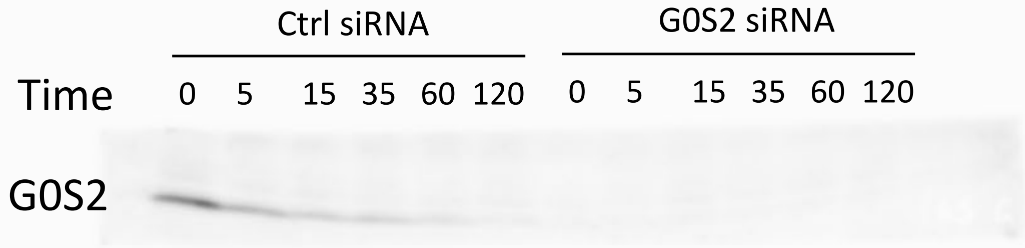

Full unedited gel for Figure 8B

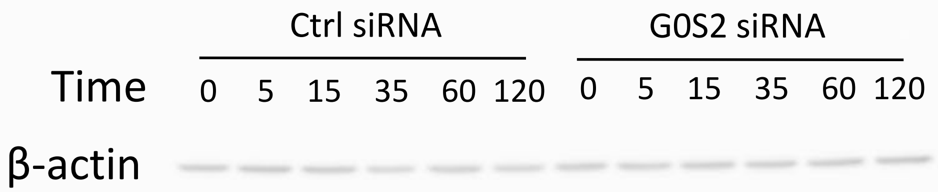

Full unedited gel for Figure 8C

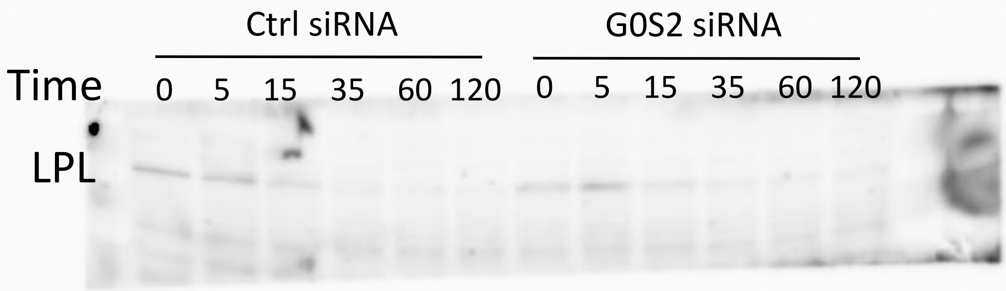

Full unedited gel for Figure 8C

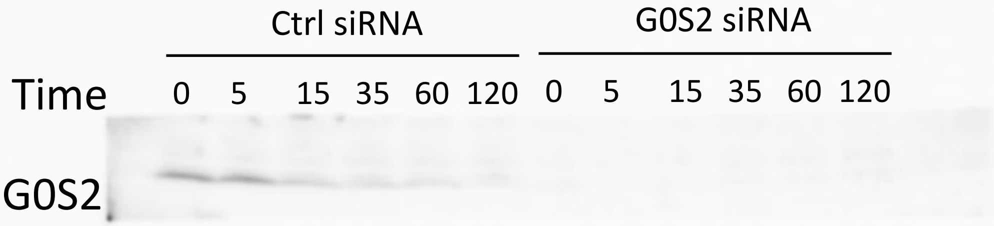

Full unedited gel for Figure 8C

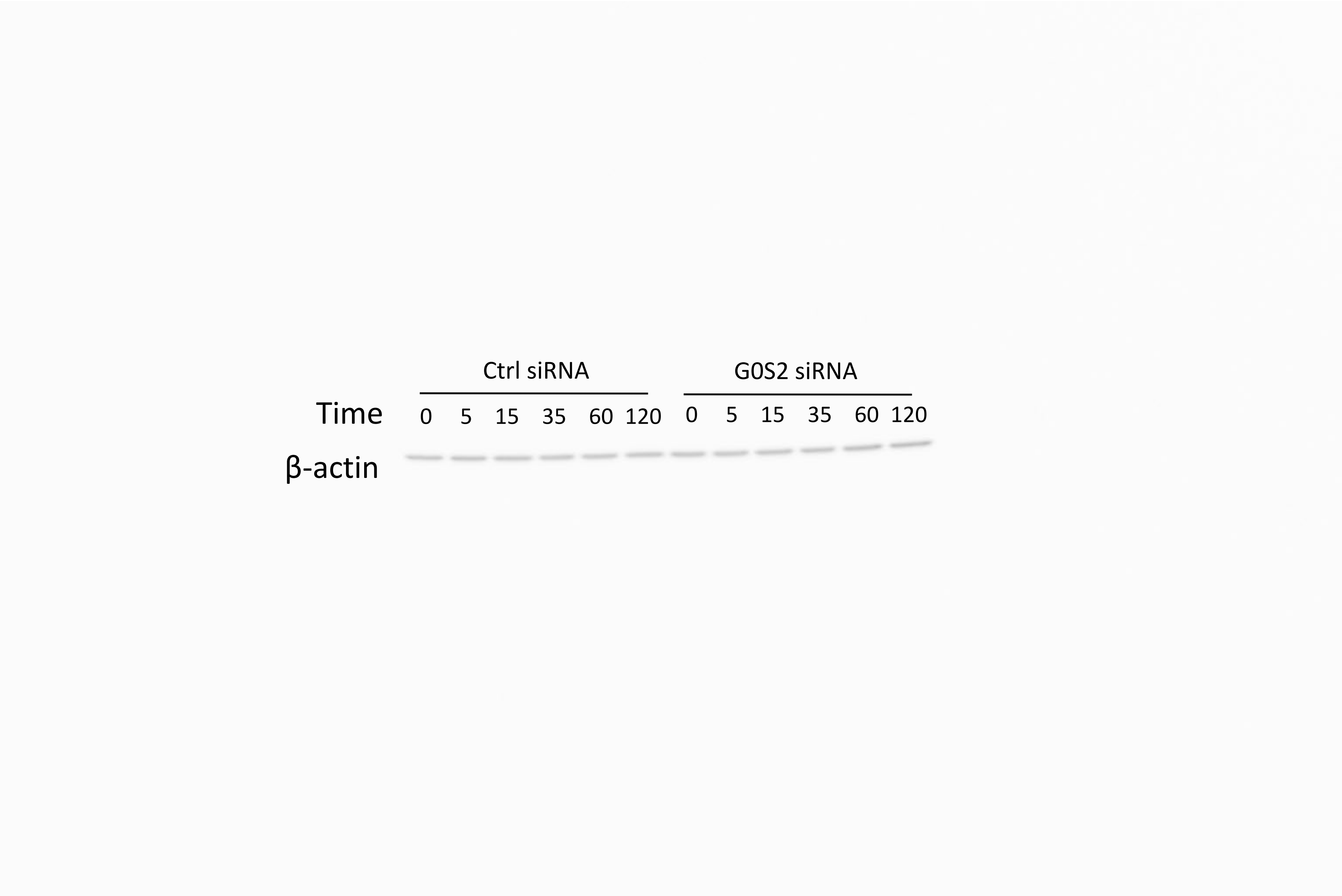

Full unedited gel for Figure 8G

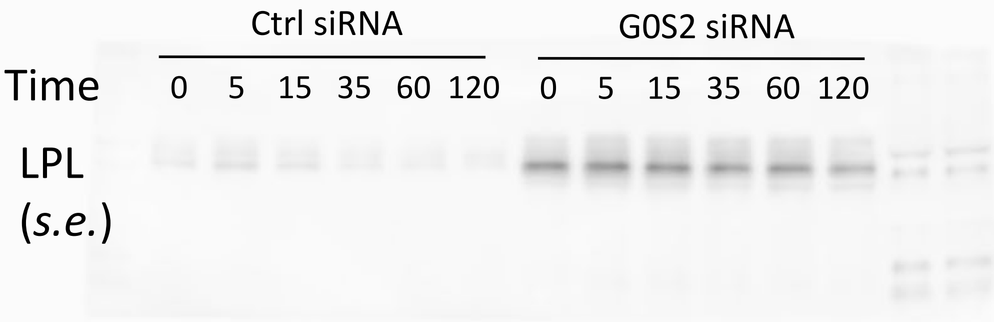

Full unedited gel for Figure 8G

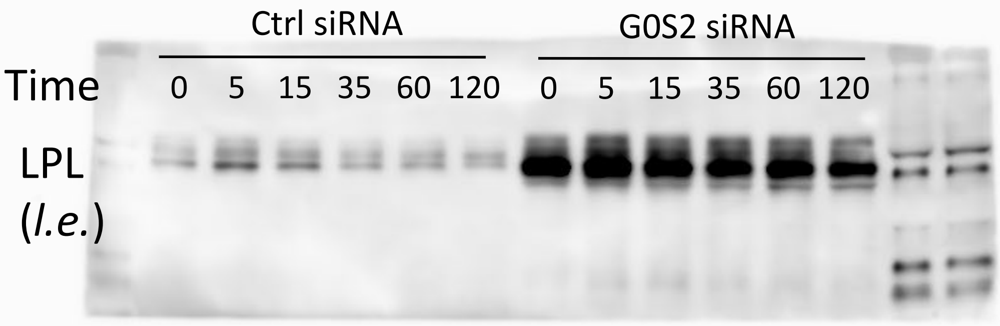

Full unedited gel for Figure 8G

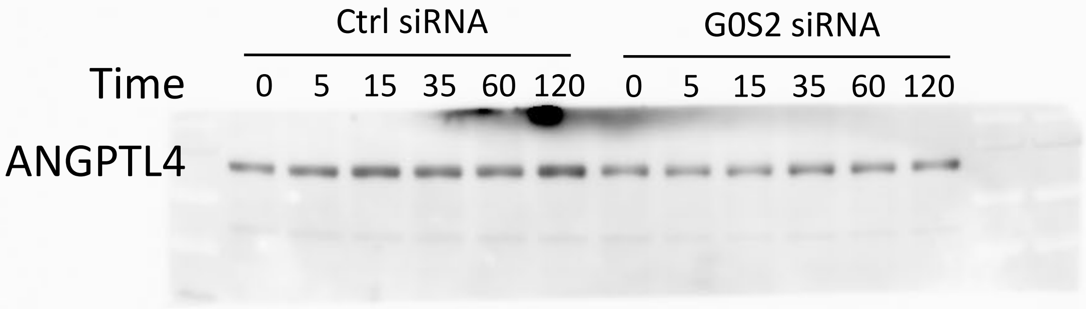

Full unedited gel for Figure 8G

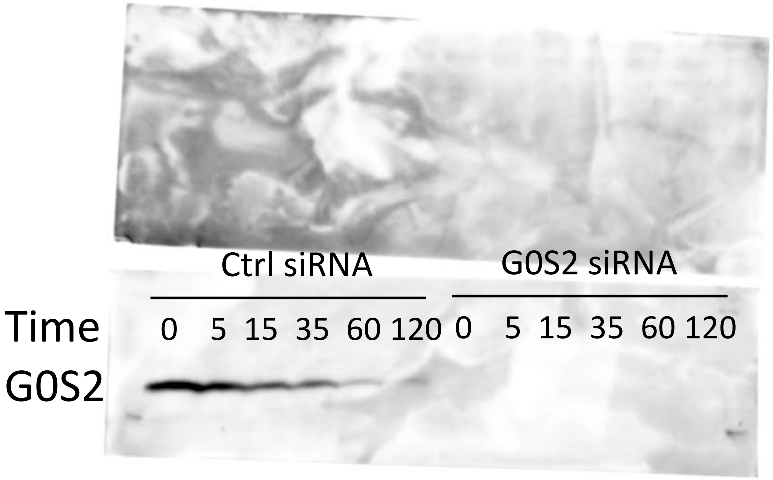

Full unedited gel for Figure 8G

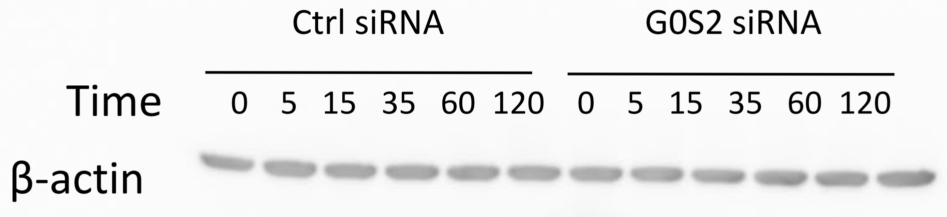

Full unedited gel for Figure 8G

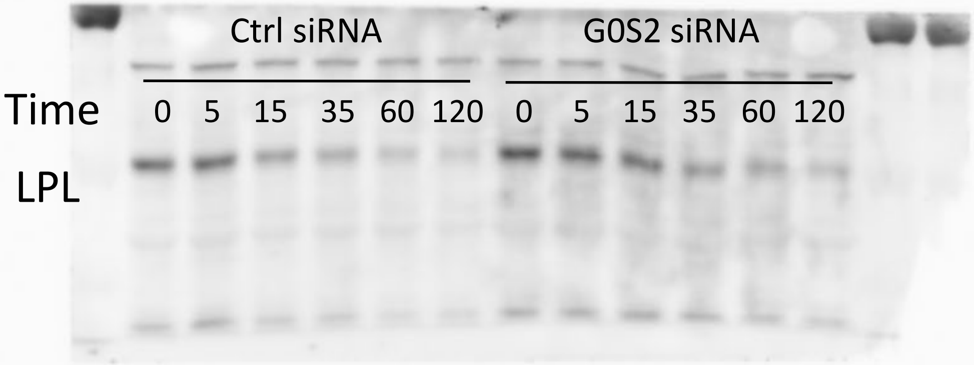

Full unedited gel for Figure 8G

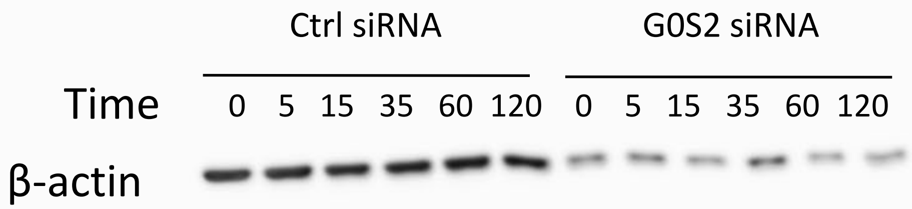

Full unedited gel for Figure S1E

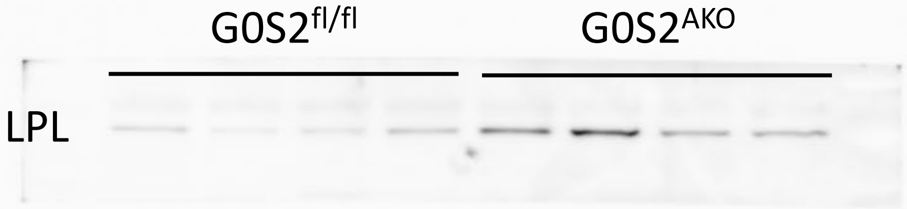

Full unedited gel for Figure S1E

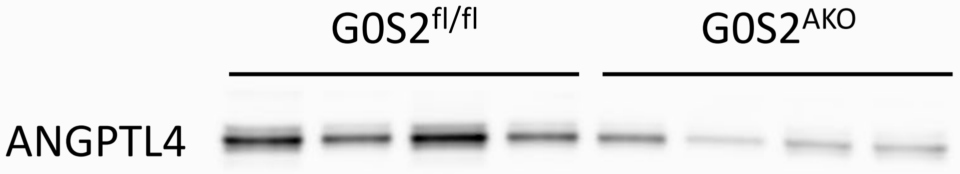

Full unedited gel for Figure S1E

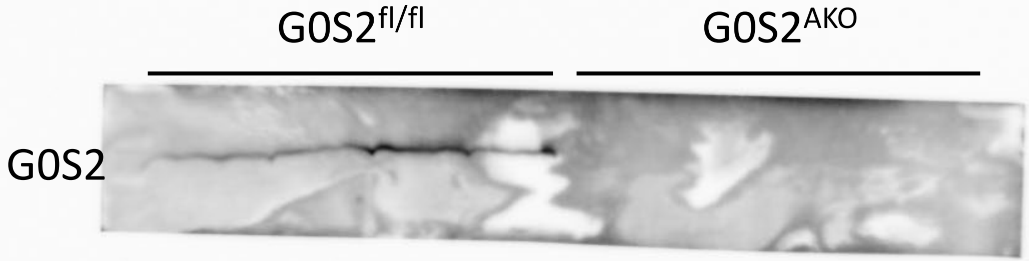

Full unedited gel for Figure S1E

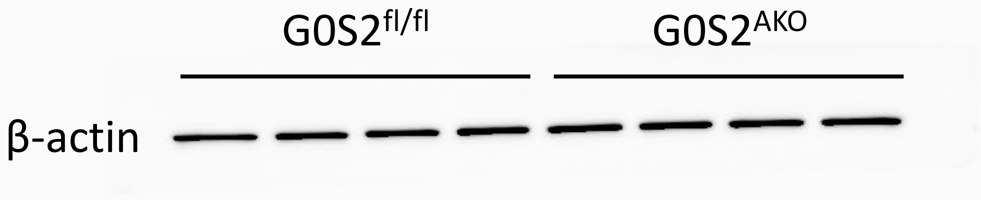

Supplement: Unedited blot and gel images [file jci-135-181754-s193.pdf]
